# Disuse‐induced muscle‐type specific alterations and adiponectin pathway response in male mice

**DOI:** 10.14814/phy2.70602

**Published:** 2025-10-20

**Authors:** Szczepanski Sébastien, Limpens Maëlle, Jenart Vincianne, Declèves Anne‐Emilie, Legrand Alexandre, Tassin Alexandra

**Affiliations:** ^1^ Laboratory of Respiratory Physiology, Pathophysiology and Rehabilitation, Research Institute for Health Sciences and Technology University of Mons Mons Belgium; ^2^ Department of Metabolic and Molecular Biochemistry, Research Institute for Health Sciences and Technology University of Mons Mons Belgium

**Keywords:** adiponectin pathway, disuse muscle atrophy, myofiber‐type

## Abstract

Disuse‐mediated Muscle Atrophy (DMA) causes persistent muscle weakness, limiting exercise training as a treatment. Adiponectin (ApN) emerged as a therapeutic candidate for muscle disorders. However, the effect of DMA on the ApN pathway remains poorly studied. Given ApN's metabolic effects, examining the ApN pathway response to disuse in relation with muscle type is essential. To mimic DMA while avoiding confounding factors, we combined HindLimb Unloading with Immobilization (HLUI) through a device allowing mouse displacements. The effects of disuse on DMA severity were studied in the slow‐twitch *Soleus* and the fast‐twitch Tibialis anterior (*TA*) muscles, together with the ApN pathway. The *Soleus* muscle presents a moderate atrophy of type IIa myofibers, whereas the *TA* muscle is more severely affected and exhibits a type I to IIa switch. HLUI increased the hybrid I/IIa myofiber proportion in both muscles, suggesting an ongoing myofiber switch that is delayed in the *Soleus* muscle. Concomitantly, HLUI enhances ApN plasma level, modifies oligomeric form proportions, and downregulates Adiporeceptors in the *Soleus* but not in the *TA* muscle. In conclusion, HLUI is associated with a higher ApN plasma level and disturbances in oligomeric form proportions. DMA severity, myofiber switch kinetics, and adiporeceptor regulation are muscle‐type dependent.

## Graphical abstract

(LeftPanel) HindLimb Unloading combined with Immobilization (HLUI) was used to induce Disuse Muscle Atrophy (DMA) via an optimized device that allow mouse displacements along the cage. As a result of muscle positioning during immobilization, the *Soleus* muscle was stretched and the *Tibialis anterior (TA)* muscle was shortened. (Right Panel) Fiber‐type‐specific effects of HLUI were studied in both muscles, along with the ApN pathway. The*Soleus* shows moderate type IIa atrophy (↓CSA, in green); *TA* displays a more severe atrophy in type IIa (↓CSA, in green) and IIb (↓CSA, in orange) fibers, as well as a type I to IIa shift (↓I, in red). Hybrid I/IIa fibers increase in both muscles (↑I/IIa). HLUI elevates ApN plasma level (↑ApN), alters ApN oligomeric forms (↓LMW, ↑MMW), and downregulates adiporeceptors in the *Soleus* (↓AdipoR1, ↓AdipoR2), but not in the *Tibialis anterior (TA)* muscle.

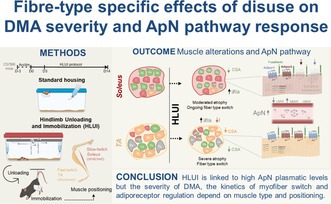

## 1 INTRODUCTION

Skeletal muscle deconditioning is a decline in muscle function that can result from a pathological condition by itself (cancer, metabolic disorders, traumas, and hereditary muscular dystrophies) or be secondary to the associated hypo‐ or inactivity, notably due to prolonged bed rest, limb immobilization, mechanical ventilation, or wheelchair dependence (Atherton et al., 2016; Gao et al., 2018; Nunes et al., 2022). In conditions of muscle hypoactivity, skeletal muscle deconditioning leads to the development of Disuse Muscle Atrophy (DMA) at the tissue level. DMA is mainly characterized by a decrease in myofiber cross‐sectional area (CSA) and a slow‐to‐fast myofiber type switch (Baehr et al., 2022; Bodine, 2013; Shenkman, 2016; Wang et al., 2017). At the molecular level, DMA is associated with a protein synthesis/degradation imbalance, abnormal oxidative stress, and mitochondrial dysfunction (Baehr et al., 2022; Baldwin et al., 2013; Bodine, 2013; Puthucheary et al., 2010; Shenkman, 2016; Vilchinskaya et al., 2018). Importantly, morphological alterations are associated with functional impairments and persistent muscle weakness, even after muscle reconditioning programs in an important group of patients (Boelens et al., 2022; Cuthbertson et al., 2010; Dos Santos et al., 2016). Moreover, DMA was suggested to be associated with impaired adult myogenesis that could limit the effect of muscle reconditioning as well as muscle regeneration in case of injury (Boelens et al., 2022; Matsuba et al., 2009).

To mimic a reversible DMA, Hindlimb Unloading (HLU) and Hindlimb Immobilization are widely used in rodents (Bodine, 2013; Marzuca‐Nassr et al., 2019; Morey‐Holton et al., 2005). However, experimental conditions in those models are often associated with reduced social interactions, stress, and body weight loss, introducing confounding factors (Moustafa, 2021; Tousen et al., 2020). In some studies, the HLU device was adapted to allow mouse displacement with forelimbs (Marzuca‐Nassr et al., 2019), but this procedure is associated with hindlimb residual movements that may limit atrophy development. Since hindlimb immobilization, when combined with HLU, has been reported to induce a more severe atrophy than HLU alone (Du et al., 2011), we used this strategy to avoid residual movements. The model optimized here was never applied to study DMA molecular mechanisms or potential treatments.

To counteract the development of DMA, most studies interrogated the effect of nutritional supplementation with antioxidant cocktails. Unfortunately, such strategies failed in human studies, and the only effective treatments remain mobilization, electrostimulation, and exercise training (ET) (Arc‐Chagnaud et al., 2020; Boelens et al., 2022). Unfortunately, patient intolerance to ET often limits muscle reconditioning, thus stressing the need for the development of pharmacological approaches to protect muscles in this frequent pathological context (Boelens et al., 2022).

At the physiological state, skeletal muscle is at the center of an inter‐organ crosstalk involving Adiponectin (ApN, encoded by the *Adipoq* gene). This adipo/myokine has autocrine, paracrine, and endocrine actions (Amin et al., 2010; Krause et al., 2008, 2019). If ApN is mainly secreted by adipose tissue, skeletal muscle is also a source and a target tissue of ApN (Krause et al., 2008). ApN post‐translational modifications result in multimeric forms found in the plasma and classified as Low (LMW), Medium (MMW), and High molecular weight (HMW) (Wang et al., 2008). Those circulating forms target tissues via ADIPOR1 and ADIPOR2 receptors, predominantly expressed in skeletal muscle and liver, and activating AMPK and PPARα pathways, respectively (Iwabu et al., 2010; Krause et al., 2008; Yamauchi et al., 2014). More recently, T‐cadherin was identified as an ApN co‐receptor required to maintain ApN at the tissue membrane (Denzel et al., 2010; Matsuda et al., 2015; Tanaka et al., 2019). As reviewed in (Abou‐Samra, Selvais, Dubuisson, & Brichard, 2020), oligomeric ApN form proportion was reported to influence ApN biological effect, but this feature was mainly studied in type 2 diabetes. In this context, HMW ApN forms are notably reported as the most biologically active through their more potent ability to increase insulin sensitivity (Basu et al., 2007; Hara et al., 2006; Pajvani et al., 2004). In addition to its well‐described anti‐diabetic, anti‐apoptotic, and anti‐oxidative properties (Kadowaki et al., 2006; Liu et al., 2015; Tian et al., 2012), ApN exerts myoprotective effects, notably by activating the ADIPOR1/AMPK/SIRT1/PGC1α axis, allowing for reduced inflammatory and oxidative stress and enhanced oxidative metabolism (Abou‐Samra, Selvais, Boursereau, et al., 2020; Abou‐Samra, Selvais, Dubuisson, & Brichard, 2020; Iwabu et al., 2010; Jiang et al., 2019). Those discoveries are mainly based on studies in mice demonstrating that ApN‐KO muscles are more sensitive to oxidative stress, inflammation, and apoptosis (Liu et al., 2015), and that muscle‐specific Adipor1‐KO mice show reduced endurance, type I fiber number, mitochondrial content, and oxidative stress‐detoxifying enzymes (Iwabu et al., 2010). Furthermore, ApN was reported to have pro‐myogenic effects (reviewed by (Abou‐Samra, Selvais, Dubuisson, & Brichard, 2020)). Notably, elastases produced by M1 macrophages at the lesion site cleave ApN into its globular form (gApN) (Waki et al., 2005), which regulates satellite cells (SC) activation and later regeneration steps (Fiaschi et al., 2009, 2012, 2014; Gamberi et al., 2016). The T‐cadherin co‐receptor (Obata et al., 2018; Tanaka et al., 2019) and ADIPOR1 (Iwabu et al., 2010) are also reported as key actors in ApN‐mediated muscle regeneration.

However, discrepancies remain about ApN roles in skeletal muscle. Indeed, in elderly patients, muscle mass loss was associated with elevated ApN circulating levels, a concept named “ApN paradox” (Baker et al., 2019; Walowski et al., 2023).

On the other hand, the therapeutic potential of ApN agonists was assessed in different models of muscle disorders, for example, *mdx* mice (a model of Duchenne Muscular Dystrophy) (Abou‐Samra, Selvais, Boursereau, et al., 2020), aged mice (Balasubramanian et al., 2022), and dexamethasone‐induced atrophy (Singh et al., 2017). In those studies, ApN pathway activation limited oxidative stress and inflammation in *mdx* mice, promoted oxidative metabolism in both aged and *mdx* mice (Abou‐Samra, Selvais, Boursereau, et al., 2020; Balasubramanian et al., 2022) and reduced atrophy induced by dexamethasone *in vivo* (Singh et al., 2017). These data support the therapeutic potential of ApN pathway activators in muscle diseases, particularly when associated with oxidative stress and metabolic alterations. However, ApN agonists have never been considered in the pathological context of DMA. Moreover, it is still unclear whether muscle disuse can affect ApN pathway components, as only one study interrogates this pathway in HLU mice. In this model, Goto et al. highlighted a down‐regulation of *Adipor1* but not of *Adipor2* mRNA levels in the *Soleus* muscle and a positive relation between muscle weight and *Adipor1* mRNA level in a suspension‐recovery experiment, suggesting that mechanical loading might regulate the expression of ApN pathway molecular actors (Goto et al., 2013). However, potential changes in Adipor1 and Adipor2 protein levels were not addressed in this study. In addition, given the metabolic roles of ApN pathway, its response to disuse needs to be better characterized in both slow‐ and fast‐twitch muscles. This point is particularly of interest when considering that many muscle disorders are associated with fiber‐type‐specific changes (Ciciliot et al., 2013; Tobias & Galpin, 2020).

In this study, we optimized a murine model of Hindlimb Unloading and Immobilization to determine the role of fiber‐type composition on the effect of muscle disuse, comparing the slow‐twitch *Soleus* and the fast‐twitch *TA* muscles. Concomitantly, we decipher variations of ApN plasma level, oligomeric form proportion, and ApN (co‐)receptor expression at the mRNA and protein levels.

## 2 MATERIALS AND METHODS

### 2.1 Animals

All animal experiments met the Belgian national standard requirements regarding animal care and were conducted in accordance with the Ethics and Welfare Committee of the University of Mons (LE023/03).

**FIGURE 1:**
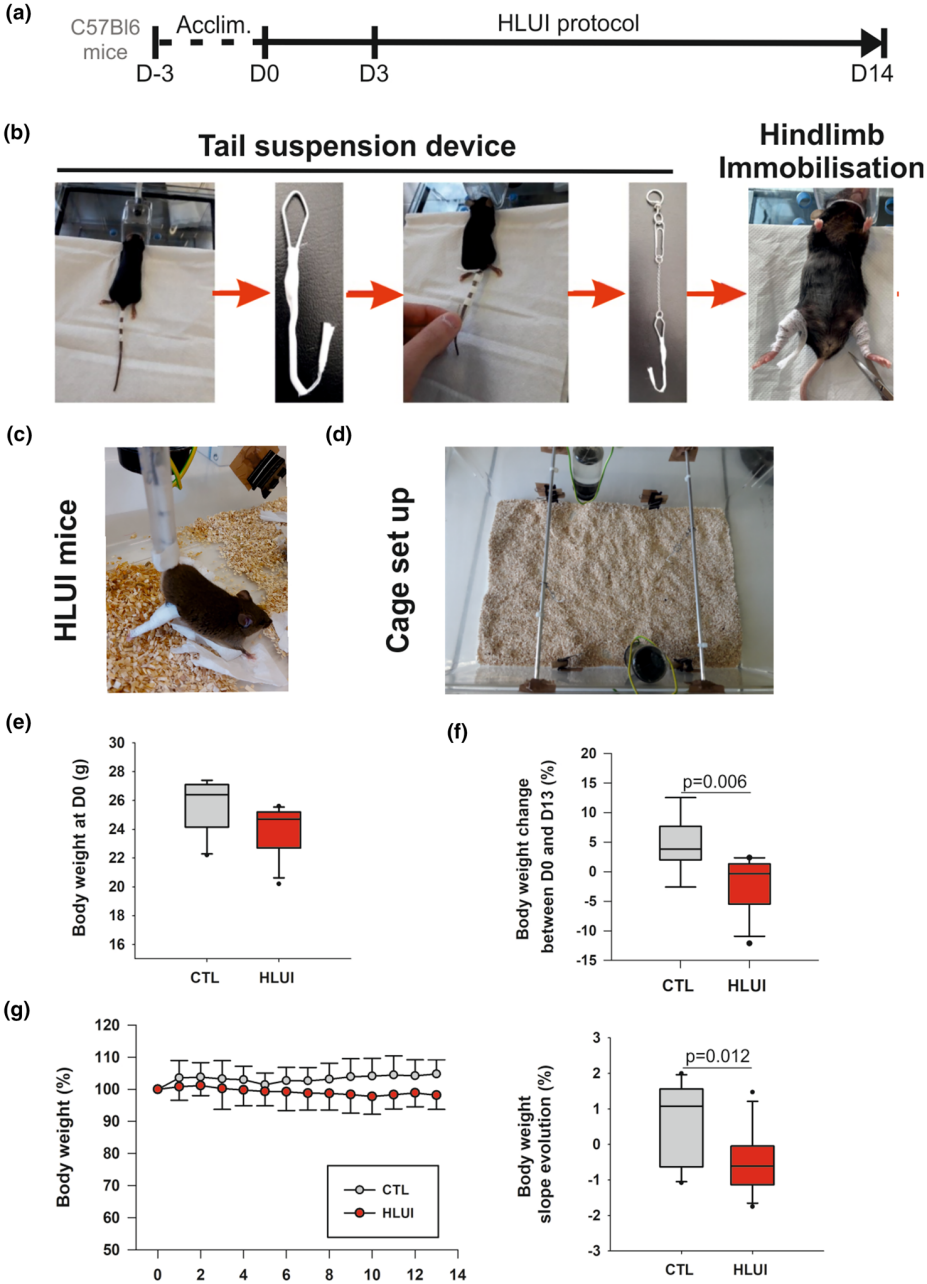
Murine model of Hindlimb Unloading and Immobilization (HLUI). (a) Timeline. During a 3‐day acclimatization period (Acclim., D‐3 to D0), mice were attached to the tail‐suspension system without any suspension. Mice were kept at floor level to allow full limb use. This step was followed by 3 (D3) or 14 days (D14) of HLUI. (b) HLUI procedure. Three bands of medical tape were fixed to the tail. A modified paperclip forming a hook was attached to the tail by using 3 other bands of medical tape. The hook was connected to a metal chain ending in a ring. Hindlimbs were immobilized in extension position, with the ankle fixed in dorsiflexion position using medical tape. HLUI mice were then suspended at a 30° angle. (c) HLUI mice. (d) Cage set up. Large rat cages were converted by adding a rod at the top. For tail suspension, the chain was attached to the rod via the ring, enabling a sliding mechanism. Mice could move along the rod axis by using their forelimbs. Two mice were housed per cage to ensure social interactions. (e, f) Body weight (b.w.) comparison in HLUI and CTL groups. (e) B.w. at D0. Data presented as boxplots; groups compared using a Mann–Whitney Rank Sum test (f) B.w. change between D0 and D13. Data presented as boxplots; groups compared using a Student's *t*‐test. (g) B.w evolution. (Left) Daily measurements normalized to b.w. at D0 (defined as the 100% baseline). Data presented as mean ± SEM. (Right) B.w slope evolution (from D1 to D13). Data presented as boxplots; groups compared using a Student's *t*‐test. CTL group: *N* = 10, HLUI group: *N* = 11.

At 12 weeks of age, male C57BL/6 mice (RRID: MGI:2159769, *Charles River*, France) were housed in large rat cages (58 × 40 × 20 cm) equipped (HLUI group) or not (CTL group) with a device allowing hindlimb unloading through tail suspension. HLUI mice were connected to this device for a 3‐day acclimatization period but kept at floor level to allow movements with both forelimbs and hindlimbs (from D‐3 to D0). Mice of the HLUI group were then tail‐suspended and hindlimbs immobilized for 3 (D3) or 14 days (D14). (Figure 1a). Relative humidity was maintained at 35%–40% with a constant room temperature (21°C) and a 12 h/12 h day/night light cycle. Animals had access to food (Carfil, maintenance dry food (RN‐01‐20 K12) and water *ad libidum*).

### 2.2 Hindlimb unloading and immobilization (HLUI)

The HLU device, adapted from Marzuca‐Nassr et al. (Marzuca‐Nassr et al., 2019), is composed of a rod fixed to the top of the cage, a pulley with a hook, and a small chain to connect the hook to a paperclip fixed on the mouse tail by using medical tapes (Leukosilk®; 1.5 × 0.5 cm). Hindlimb muscles were immobilized in an extended position from the ankle (which is in a dorsiflexion position) to the upper hip using medical band‐aids (Nepenthes; 16 × 1 cm) (Figure 1b). This system allows mice to be suspended at a 30° angle by maintaining mouse movement with their forelimbs in the rod axis (Figure 1c,d). Two mice were housed in a cage to allow social interactions.

### 2.3 Muscle collection and preparation

After 14 days of protocol, the *Soleus* and *Tibialis anterior* (*TA*) muscles were collected to perform morphometrical analyses and molecular investigations (RT‐qPCR and western blots). Morphometrical analyses required muscle embedding in OCT cryo‐compound (ImmunoLogic, 1620‐C) frozen in isopentane cooled with liquid nitrogen. Cryosections (8 μm) were performed with a cryotome (Leica CM1950). Contralateral muscles were snap‐frozen in liquid nitrogen to allow molecular investigations. Blood was collected and centrifuged (13,500 rpm, 15 min) to isolate plasma for ELISA assays. Muscles were also collected at day 3, as *Fbxo32* (encoding Atrogin‐1) overexpression was anticipated to occur at early timepoints, before atrophy installation (Atherton et al., 2016; Bodine et al., 2001; Bodine & Baehr, 2014). Those data are provided as Supporting Information.

### 2.4 Morphometrical analyses in hindlimb muscles

#### 2.4.1 Myofiber type immunofluorescence staining

*Soleus* and *TA* muscles cryosections were blocked for 1 h at room temperature with 10% Goat‐serum/PBS (VWR, S2000‐100) before being incubated for 2 h at room temperature with a primary antibody cocktail directed against Myosin Heavy Chain 7 (MyHC7) (type I fibers, IgG2b, clone BA‐D5, 1:50, DSHB, RRID: AB_2235587), MyHC2 (type IIa fibers, IgG1, clone SC‐71, 1:100, DSHB, RRID: AB_2147165), MyHC4 (type IIb fibers, IgM, clone BF‐F3, 1:10, DSHB, RRID: AB_2266724), and laminin (rabbit IgG, ab 11,575, 1:50, Abcam, RRID: AB_298179). Slides were washed 3 times in PBS and incubated for 1 h with secondary antibodies directed against mouse IgG2b (Alexa 647 anti‐mouse IgG2b, A‐21242, 1:100, Thermofisher), mouse IgG1 (Alexa 488, anti‐mouse IgG2b, A‐21121, 1:100, Thermofisher), mouse IgM (Alexa 555, anti‐mouse IgM, A‐21426, 1:50, Thermofisher), and Rabbit IgG (Alexa 405 anti‐rabbit IgM, ab17652, 1:50, abcam) to label respectively type I (Cy5 channel), type IIa (FITC channel), and type IIb (TRITC channel) myofibers as well as laminin (DAPI channel). Unstained myofibers are commonly considered type IIx myofibers, as described by (Bloemberg & Quadrilatero, 2012). Slides were washed 3 times in PBS and mounted with ProLong™ Gold Antifade Mountant (P36934, Invitrogen). Images were then captured to cover the whole muscle section with a Nikon Eclipse i80 microscope (10x magnification).

#### 2.4.2 Images processing and measurements

Each capture was processed with *QuPath‐0.5* software to subtract the background fluorescence signal and provide DAPI/Cy5, DAPI/FITC, DAPI/TRITC, and merged images. Processed individual images were then stitched with *Image Composite Editor* software to reconstitute the whole muscle section in the 4 cited channel combinations without pixel down‐sampling. Reconstituted muscle sections were then segmented thanks to the *Cellpose v2.2.3* software (CPx pre‐trained model) (Pachitariu & Stringer, 2022; Stringer et al., 2021) to determine the Regions Of Interest (ROIs) corresponding to positive myofibers in the channel combination of interest. Since *Cellpose v2.2.3* allows exporting ROI as a mask.PNG image, we used the LabelsToRoi plugin (Waisman et al., 2021) to transpose myofiber segmentation in the *Fiji* software for ROI measurements. Each myofiber CSA was finally obtained after conversion of the corresponding ROI (in pixels) in μm^2^ with the pixel/μm ratio. Myofibers were then classified into clusters according to their area (<120 μm^2^, 120–300 μm^2^, and every 300 μm^2^ until 3900 μm^2^ in the *Soleus* muscle and 6000 μm^2^ in the *TA* muscle) to evaluate changes in myofiber CSA distribution. Minimum Feret's diameter (MFD), a geometrical parameter used for morphometric analysis, was also measured from the segmented ROIs as it is less affected by the orientation of the muscle section (Briguet et al., 2004).

Double positive (MyH7^+^/MyH2^+^) myofibers were identified by overlapping DAPI/Cy5 images Cellpose segmentation (corresponding to type I fibers) on DAPI/FITC images (corresponding to type IIa fibers) with the LabelsToROI plugin of *Fiji*. The percentage of double‐positive myofibers was expressed as the percentage of MyH7^+^ myofibers that are also MyH2^+^.

### 2.5 RT‐qPCR analyses

Total RNAs were extracted from *Soleus* and *TA* muscles with Trizol reagent (Invitrogen, 155,596–026) according to the manufacturer's guidance before being treated with DNAse I (ThermoFischer, 18,068–015). cDNAs were synthesized from 1 μg of RNA using the Maxima First Strand cDNA synthesis kit (ThermoFischer, K1641). RT‐qPCRs were performed in triplicate for each primer (Eurogentec) (Table S1) with the SYBR Green FastStart Essential DNA Green Master (Roche, 0640271001) and by using the LightCycler®96 (Roche) device (cycling conditions: initial denaturation step at 95°C for 10 min, followed by 40 cycles of 15 s at 95°C and 60 s at primer Tm). Raw data were analyzed with the LightCycler®96 software and quantified by using the 2^−ΔΔCt^ method (*Rplp0* as the housekeeping gene, data normalized to CTL).

### 2.6 ApN plasma components

ApN plasma concentrations were measured by using the Adiponectin/Acrp Quantikine ELISA kit (R&D, MRP300) according to the manufacturer's instructions. Relative amounts of LMW, MMW and HMW ApN circulating forms were determined using a non‐denaturating SDS‐PAGE electrophoresis followed by a western blot. Based on ApN plasma concentrations provided by ELISA assay, 10 ng of ApN were separated onto an 8% polyacrylamide gel (125 V; 2 h20) before being transferred onto a nitrocellulose membrane (Amersham). Membranes were then stained with Ponceau Red, washed 3 times in TBS‐Tween (0.2%), and blocked 1 h in 5% non‐fat dry milk diluted in TBS‐T. Primary antibodies directed against ApN (1:1000, Rb IgG Ab85827, Abcam, RRID:AB_10675534) as well as corresponding secondary HRP conjugated antibodies (Donkey anti‐Rb IgG, VWR, NA934) were diluted in 1% non‐fat dry milk TBS‐T for incubation for 2 h at 4°C and for 1 h at room temperature, respectively. The HRP signal was visualized using Supersignal West Femto Max Sensitivity Kit (Thermo Fisher Scientific, 34,095). Densitometry was performed using *Fiji* software. The densitometry signal was normalized to the total proteins stained by Ponceau Red. The signal corresponding to LMW, MMW, and HMW ApN forms was then normalized on the total ApN signal and therefore expressed as a percentage. S_A_ Index, commonly used as an indicator of insulin sensitivity, corresponds to the ratio HMW/(LMW + MMW + HMW) (Pajvani et al., 2003).

### 2.7 Western blot analyses

*Soleus* and *TA* muscles were homogenized in lysis buffer (CelLytic, Sigma, C3228; Protease Inhibitor Cocktail, Sigma, P8340; Phosphatase Inhibitor Cocktail, Millipore, 524,632). Equal protein amounts were then separated on a 12% SDS‐PAGE gel (100 V; 3 h 30) before being transferred onto a nitrocellulose membrane (Amersham) by using the Trans‐Blot Turbo Transfer System (Biorad™). Membranes were then stained with Ponceau Red, washed 3 times in TBS‐Tween (0.2%), and blocked 1 h in 5% non‐fat dry milk diluted in TBS‐T. Primary antibodies were directed against Adipor1 (1:1000, overnight at 4°C, Rb IgG, Gentaur, ADIPOR12‐A), AdipoR2 (1:1000, overnight at 4°C, Rb IgG, Gentaur, ADIPOR22A), and T‐cadherin (1:1000, overnight at 4°C, R&D, AF3264). After 3 washes, membranes were incubated with corresponding secondary HRP‐conjugated antibodies (Donkey anti‐Rb IgG, 1:5000, 1 h at room temperature, VWR, NA934;Rabbit anti‐goat IgG, 1:5000, 1 h at room temperature, Abcam, ab6741). All antibodies were diluted in 1% non‐fat dry milk TBS‐T. The HRP signal was visualized using the Supersignal West Femto Max Sensitivity Kit (Thermo Fisher Scientific, 34,095) and the Fusion FX7 spectra (Vilber, France). Densitometry was performed using the *Fiji* software. The densitometry signal was normalized to the total proteins stained by Ponceau Red.

### 2.8 Statistical analyses

Statistical analyses were done using Sigma Plot software, version 14. For comparison, depending on normality and equal variance tests, we used: (i) a Student's *t*‐test (plasma ApN level and ApN oligomers, *AdipoQ* and *Fbxo32* in the *Soleus*, adipo(co)receptor mRNA and protein levels; myofiber CSA, proportion and MFD as indicated in figure legends), or (ii) a Welch's *t*‐test (*Adipoq* mRNA, type IIx CSA and MFD in the *TA* muscle), or (iii) the non‐parametric Mann–Whitney Rank Sum test (Type I myofiber CSA and type IIb myofiber proportion in the *Soleus* muscle), or (iv) a Chi‐square test (myofiber CSA distribution in the *Soleus* and *TA* muscles). Mouse body weights (b.w.) at D0 were compared with the non‐parametric Mann–Whitney Rank Sum test. The change of b.w. between D0 and D13 was compared with a Student's *t*‐test. B.w evolution during the protocol was also evaluated using linear regression, calculation of slope coefficients, and mean comparison between HLUI and CTL mice with a Student's *t*‐test.

According to normality and equal variance test results, the graphical representations were performed as follows. Myofiber size distributions were expressed as mean ± SD and represented as histograms. Myofiber type proportions were represented as stacked histograms. Mouse b.w. slope evolution, CSA, MFD, percentage of hybrid fibers, *Adipoq* mRNA, ApN plasma level, ApN oligomer proportions, Adipor1, Adipor2, and T‐cadherin mRNA and protein levels were represented as box (median, 25th and 75th percentile) and whisker (5th and 95th percentile) plots.

## 3 RESULTS

### 3.1 Optimization of a murine model of muscle disuse

To mimic DMA in mice, we used a murine model of Hindlimb Unloading (HLU) that was adapted to limit confounding factors such as stress and consecutive body weight (b.w.) loss. To this aim, we added an acclimatization period of 3 days and used a device allowing for maintenance of social interactions and mouse displacements throughout the cage via their forelimbs. Moreover, we combined HLU with a hindlimb immobilization procedure (HLUI) to avoid residual hindlimb movements during displacements that may limit DMA development (Figure 1a,d).

We first verified the impact of this optimized HLUI procedure on b.w. evolution throughout the protocol. If HLUI mouse b.w. tended to be slightly lower in HLUI mice as compared to CTL at baseline, this difference is not significant from a statistical point of view (D0, Figure 1e). In the HLUI group, a slight reduction of b.w. was shown between D0 and D13 (−1.8 ± 4.4%), and a moderate gain was observed in the CTL group (+4.5 ± 4.6%) (Figure 1f). B.w. evolution is also presented in Figure 1g. Accordingly, mean b.w. in the HLUI group was relatively stable, with a slightly negative slope value (−0.5 ± 0.85%). In the CTL group, the small gain of b.w. results in an evolution slope of 0.7 ± 1.1%, statistically different from the HLUI group (*p* = 0.012, student's *t*‐test) (Figure 1g). Regarding mean food consumption, no differences were found between HLUI and CTL groups (Figure S1).

To further characterize the HLUI model, we also investigated the expression of *Fbxo32 (*encoding Atrogin‐1), an early marker of DMA development in rodents. We showed that 3 days of HLUI successfully induced *Fbxo32* upregulation in the *Soleus* and the *TA* muscles (Figure S2).

### 3.2 The effects of muscle disuse in mice are fiber‐type dependent

#### 3.2.1 *Soleus* slow‐twitch muscle

**FIGURE 2:**
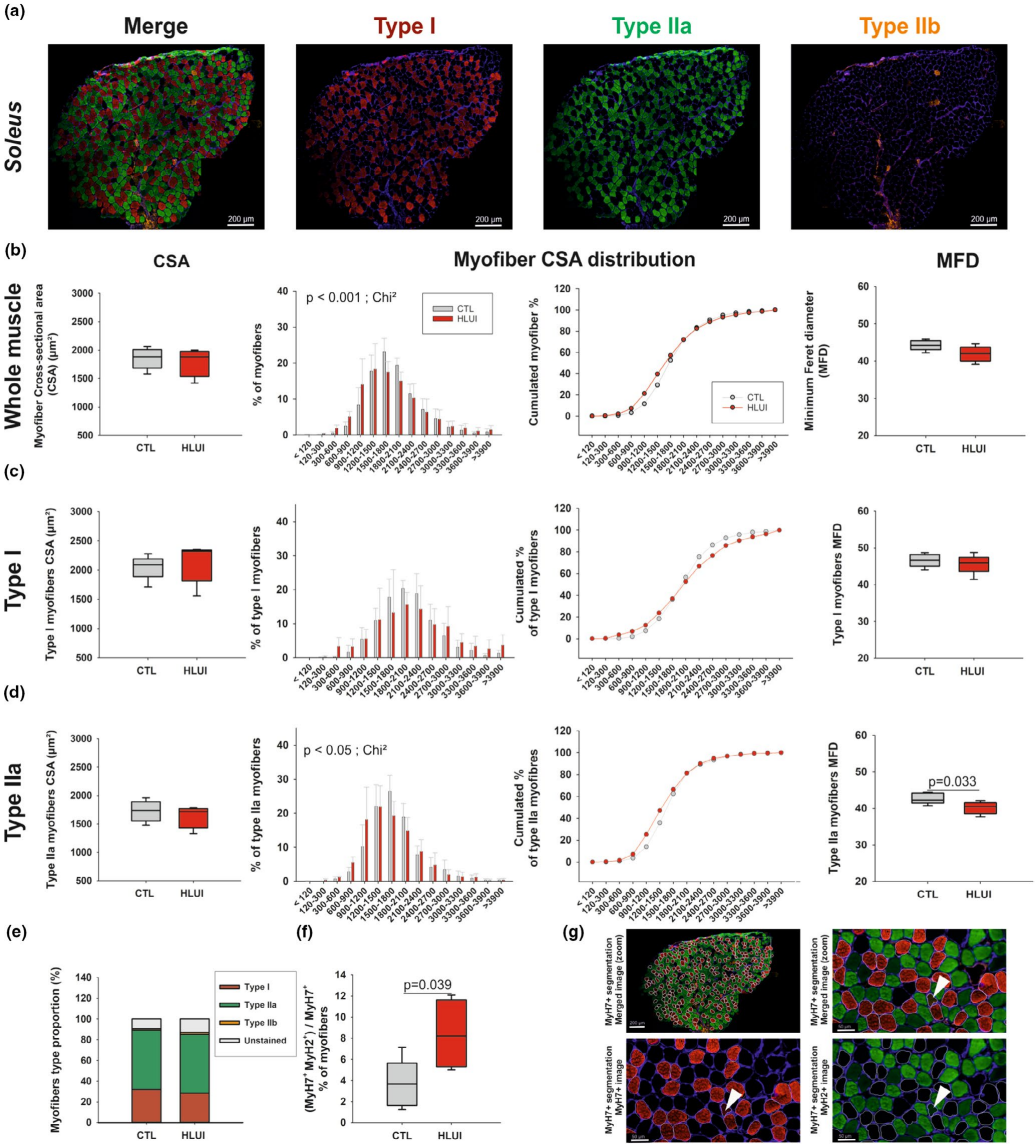
Effects of HLUI on *Soleus* muscle morphometrical parameters. (a) Type I, IIa and IIb fiber co‐immunofluorescence: Representative fields. (b–d) Cross‐sectional Area (CSA), myofiber CSA distribution, and Minimum Feret's diameter (MFD) in the whole muscle (b), in type I (c), and in type IIa myofibers (d). CSA: Data presented as boxplots; groups compared using Student's *t*‐tests (NS). Myofiber CSA distribution: Data presented as mean ± SD (left) and as cumulative percentages (right); groups compared using Chi‐square (Chi^2^) tests (*p* < 0.05, *p* < 0.001, as indicated). MFD: Data presented as boxplots; groups compared using Student's *t*‐tests (*p* = 0.033, as indicated) (e) Myofiber type proportions. Data presented as stacked bars; groups compared using a Student's *t*‐test (NS). (f) Percentage of type I myofibers also positive for IIa myofiber marker. Data presented as boxplots; groups compared using a Student's *t*‐test (*p* = 0.039, as indicated). (g) Representative field showing hybrid (I/IIa) myofiber detection. Arrows: Type I fibers (in red) also positive for type IIa marker (in green). Laminin is stained in blue. CTL group: *N* = 5, HLUI group: *N* = 5.

As expected, type I, type IIa, and type IIb myofibers were detected in CTL and HLUI *soleus* muscles (Figure 2a). Unstained myofibers, commonly considered as IIx myofibers (Bloemberg & Quadrilatero, 2012), were also observed (Figure S3).

For a precise assessment of the whole muscle CSA (Figure 2b), we measured all myofiber CSA individually and observed that HLUI did not affect the *Soleus* myofiber mean CSA. However, the analysis of myofiber CSA distribution showed an increased proportion of myofibers in smaller area clusters (<1200 μm^2^) in HLUI mice compared to CTL (*p* < 0.001, Chi‐square). To minimize potential risks of bias linked to sectioning angle orientation, the MFD was also measured, but no modification was observed in the HLUI group.

Myofiber‐type‐specific morphometrical analyses (Figure 2c,d) revealed that HLUI had no impact on type I myofiber CSA (Figure 2c). However, type IIa myofiber CSA distribution showed a switch towards fibers with smaller CSA (<1200 μm^2^) in the HLUI groups compared to CTL mice (*p* < 0.05, Chi‐square). Accordingly, type IIa myofiber mean MFD was also reduced (*p* < 0.05, Student's *t*‐test) (Figure 2d). Type IIb myofibers were only detected on 3 to 5 *Soleus* muscles when considering both groups. Although type IIb and IIx (unstained) myofiber CSA appeared not affected by HLUI, a significant reduction of type IIb myofiber MFD was observed in HLUI *Soleus* muscles (*p* < 0.05, Student's *t*‐test) (Figure S3). Taken together, our results highlight that the impact of HLUI on myofiber CSA ranges from weak to moderate, and mostly concerns type IIa myofibers.

Since DMA is commonly described as characterized by a myofiber type switch (Bodine, 2013; Wang et al., 2017), we also interrogated this feature. In the CTL group, the percentage of type I, type IIa, and type IIb myofibers in the *Soleus* muscle was 35.3 ± 8.2%; 63.1 ± 6.2%, and 1.6 ± 2.5%, respectively. These proportions were not significantly modified upon HLUI (Figure 2e). However, morphometrical analyses revealed the presence of MyH7^+^(Cy5)/MyH2^+^(FITC) double‐positive myofibers, generally named hybrid I/IIa (or switching) myofibers. Interestingly, the hybrid myofiber proportion increased in HLUI *Soleus* muscles (8.4 ± 3.3%) as compared to CTL (3.7 ± 2.3%) (*p* < 0.05, Student's *t*‐test) (Figure 2f,g), suggesting an ongoing slow‐to‐fast myofiber transition.

#### 3.2.2 *Tibialis anterior* fast‐twitch muscle

**FIGURE 3:**
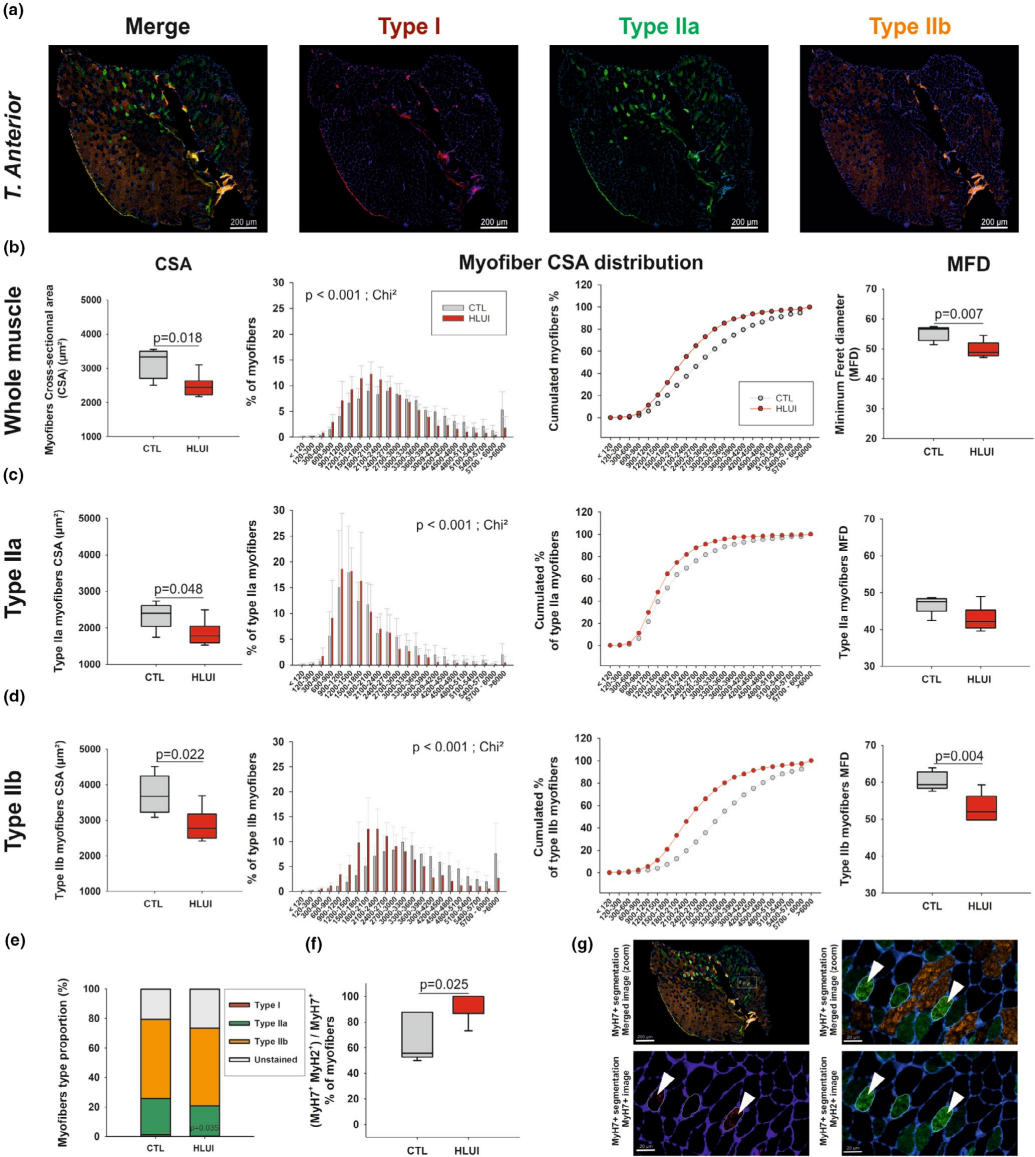
Effects of HLUI on the *Tibialis anterior* muscle morphometrical parameters. (a) Type I, IIa, and IIb fiber co‐immunofluorescence: Representative fields. (b–d) Cross‐sectional Area (CSA), myofiber CSA distribution, and Minimum Feret's Diameter (MFD) in the whole muscle (b), in type IIa (c), and in type IIb myofibers (d). Data presented as in Figure 2. CSA: Student's *t*‐tests (*p* = 0.018, *p* = 0.048, *p* = 0.022, as indicated). Myofiber CSA distribution: Chi‐square (Chi^2^) tests (*p* < 0.001, as indicated). MFD: Student's *t*‐tests (*p* = 0.007, *p* = 0.004, as indicated). (e) Myofibers type proportions: Student's *t*‐test (*p* = 0.035 for type I myofibers). (f) Percentage of type I myofibers also positive for IIa myofibers markers. Data presented as in Figure 2; Student's *t*‐test (*p* = 0.025, as indicated). (g) Representative fields showing hybrid (I/IIa) myofiber detection. Image annotated as in Figure 2. CTL group: *N* = 5, HLUI group: *N* = 5.

MyHC immunodetection in *Tibialis anterior* (TA) muscle cryosections allowed the detection of type I, type IIa, type IIb and unstained (IIx) myofibers in CTL and HLUI mice (Figure 3a). HLUI decreased the whole *TA* muscle fiber CSA, with a mean CSA of 2484 ± 328 μm^2^ in HLUI mice, and of 3147 ± 435 μm^2^ in the CTL group (*p* < 0.05, Student's *t*‐test) (Figure 3b). Accordingly, myofiber CSA distribution analyses indicated that HLUI *TA* muscles presented a higher percentage of myofibers in clusters corresponding to small/intermediate CSA (<2700μm^2^) as compared to CTL *TA* muscles (*p* < 0.001, Chi‐square). MFD measurements are also in agreement with a reduced myofiber CSA in HLUI *TA* muscle (*p* < 0.01, Student's *t*‐test) (Figure 3b).

Regarding fiber‐type‐specific changes in the *TA* muscle (Figure 3c,d), HLUI mainly affected type IIa and type IIb myofibers. Indeed, type IIa myofiber CSA was reduced in HLUI mice (1852 ± 343 μm^2^) as compared to CTL (2342 ± 367 μm^2^) (*p* < 0.05, Student's *t*‐test) (Figure 3c). Moreover, HLUI mice also presented changes in type IIa myofibers CSA distribution in favor of smaller CSA clusters (<1800 μm^2^) (*p* < 0.05, Chi‐square) (Figure 3c). Similarly, type IIb myofibers CSA was decreased in HLUI mouse *TA* (2864 ± 474 μm^2^) as compared to CTL (3724 ± 555 μm^2^) (*p* < 0.05, Student's *t*‐test) (Figure 3d), and type IIb myofibers CSA distribution in HLUI mice shifted towards small and intermediate clusters (<3000 μm^2^) (*p* < 0.001, Chi‐square) (Figure 3d). HLUI did not significantly impact type I and unstained myofiber mean CSA, but we highlighted changes in unstained fiber CSA distribution (*p* < 0.001, Chi‐square) (Figure S4).

As concerns myofiber type proportion (Figure 3e), CTL *TA* muscle presents 1.3 ± 0.8% of type I, 24.4 ± 5.3% of type IIa, 53.6 ± 9% of type IIb, and 20.6 ± 9% of unstained (IIx) myofibers. Importantly, type I myofiber percentage was found to be significantly reduced in HLUI *TA* muscles (0.4 ± 0.4%) (*p* < 0.05, Student's *t*‐test), whereas type IIa (20.5 ± 10.6%), type IIb (52.5 ± 11.8%), and type IIx (26.6 ± 8.8%) myofiber proportions were unchanged (Figure 3e). This is consistent with the analysis of double‐positive fibers (Figure 3f,g) that showed that almost the totality of MyH7^+^ (type I myofibers) in the *TA* muscle of HLUI mice (94.7 ± 11.9%) are also MyH2^+^ (type IIa myofibers). In the CTL group, the corresponding mean percentage was only 67.2 ± 18.7% (*p* < 0.05, Student's *t*‐test). Those results suggest that HLUI is accompanied by a slow‐to‐fast myofiber transition in the *TA* muscle.

### 3.3 HLUI is accompanied by an enhancement of ApN plasma level and modification of ApN oligomer distribution

**FIGURE 4:**
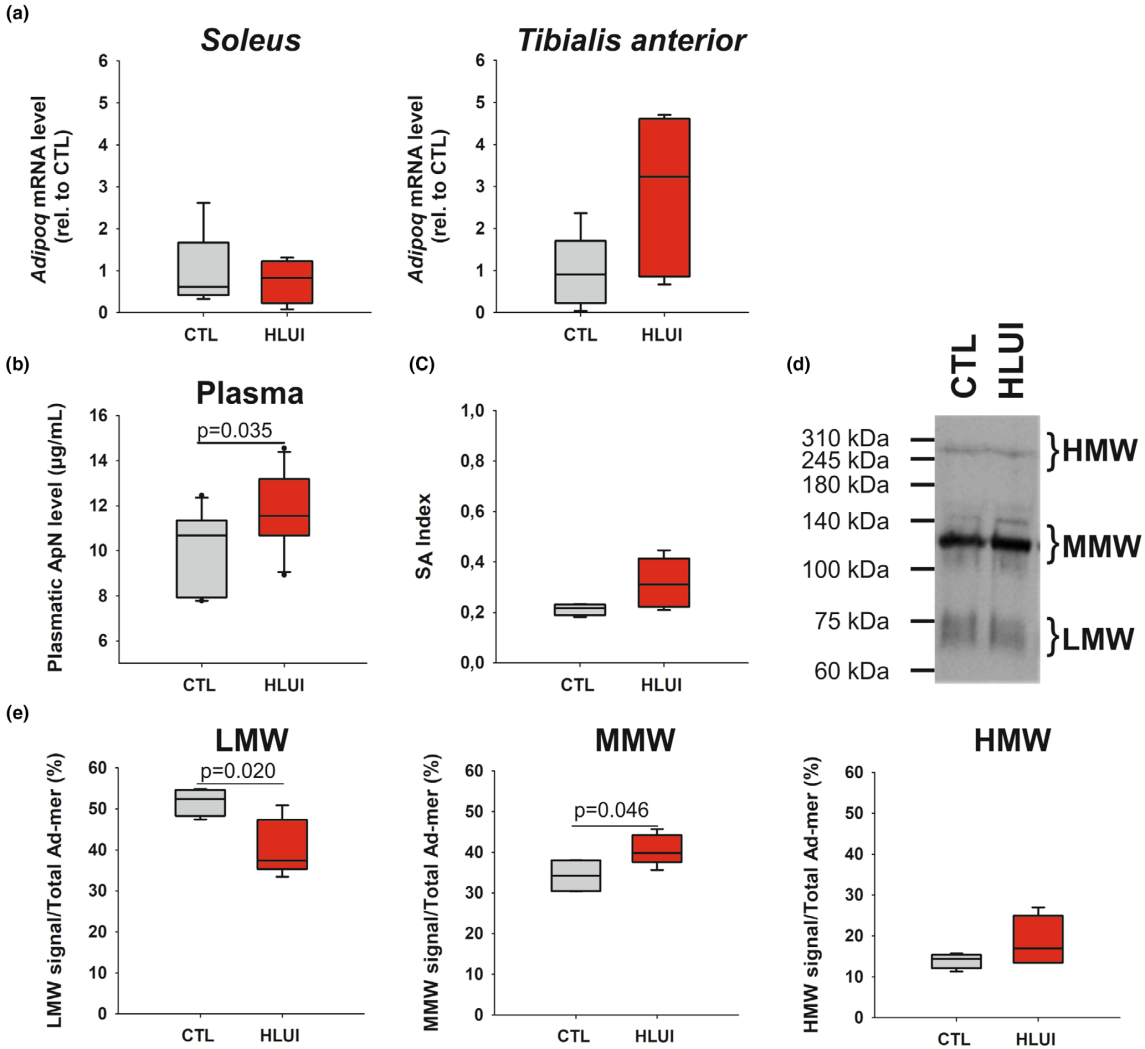
Effects of HLUI on *Adipoq* mRNA level, ApN plasmatic level, and oligomer distribution. (a) *Adipoq* mRNA levels assessed in the *Soleus* and the *Tibialis anterior* muscles by RTqPCR. Data presented as boxplots; groups compared using a Student's *t*‐test (NS). CTL group: *N* = 6, HLUI group: *N* = 5. (b) Plasma ApN levels measured by ELISA. Data presented as boxplots; groups compared using a Student's *t*‐test (*p* = 0.035, as indicated). CTL group: *N* = 10, HLUI group: *N* = 11 (c–e) Analyses of High (HMW), Medium (MMW), and Low (LMW) molecular weight ApN oligomers in the plasma using non‐denaturing PAGE‐SDS and western blot. CTL group: *N* = 4, HLUI group: *N* = 5. (c) S_A_ Index corresponds to the ratio HMW/(LMW + MMW + HMW). Data presented as boxplots; groups compared using a Student's *t*‐test (NS). (d) Representative blot. (e) ApN oligomer distribution. The signal of each oligomeric form was normalized to Ponceau Red and reported as a percentage of the total ApN signal. Data presented as boxplots; groups compared using Student's *t*‐tests (*p* = 0.020, *p* = 0.046, as indicated).

The gene expression of *Adipoq* (encoding ApN) was not significantly modified by HLUI, either in the *Soleus* or the *TA* muscles. However, we noticed that *Adipoq* expression tends to be higher but is also more variable in HLUI *TA* muscles than in the CTL group (Figure 4a).

Interestingly, ApN plasma level was increased in HLUI mice (11.73 ± 1.73 μg/mL) as compared to CTL (10.02 ± 1.70 μg/mL) (*p* < 0.05, Student's *t*‐test) (Figure 4b). Since the distribution of ApN oligomers and particularly the proportion of HMW forms (commonly calculated through the S_A_ index) are known to influence the biological effects of this adipokine, we investigated those parameters in the HLUI model (Figure 4c–e, Figure S5). However, the S_A_ index (Figure 4c) and HMW form proportion (Figure 4e) were not significantly modified by HLUI. In contrast, the proportion of LMW forms was reduced in HLUI mice (40.52 ± 6.87%) as compared to CTL (50.79 ± 3.36%) (*p* < 0.05, Student's *t*‐test) (Figure 4e). As well, the proportion of MMW ApN forms was increased (40.71 ± 3.77%) when compared with CTL (34.23 ± 4.27%) (*p* < 0.05, Student's *t*‐test) (Figure 4e).

### 3.4 The effect of muscle disuse on ApN (co)receptors expression depends on muscle type

**FIGURE 5:**
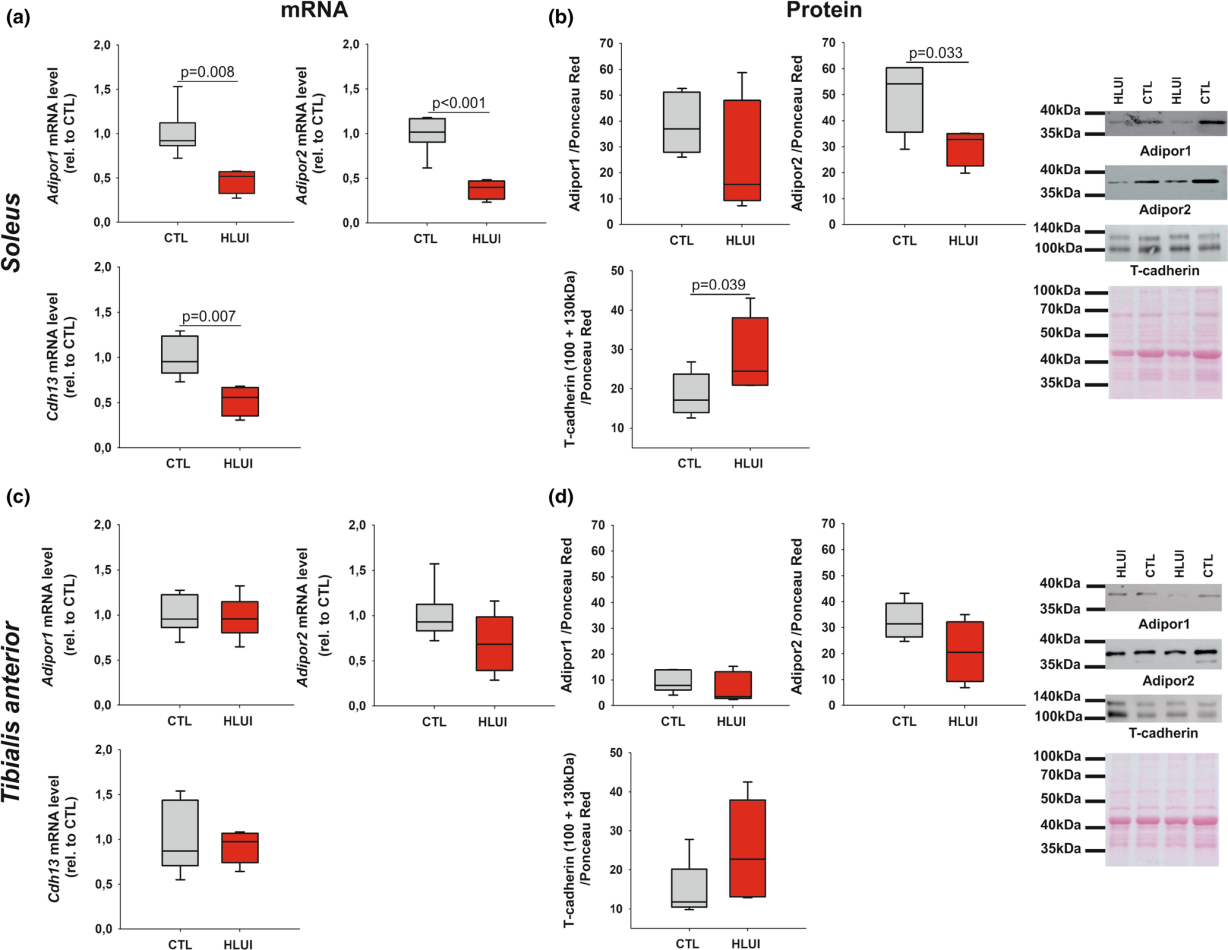
Effects of HLUI on gene expression (a, c) and protein level (b, d) of adiporeceptors in the *soleus* (a, b) and the *Tibialis anterior* (c, d) muscles. (a, c) *Adipor1, Adipor2* and *Cdh13* mRNA levels were assessed by RTqPCR. Data presented as boxplots; groups compared using Student's *t*‐tests (*p* = 0.008, *p* < 0.001, *p* = 0.007, respectively, as indicated). CTL group: *N* = 6, HLUI group: *N* = 5. (b, d) Adipor1, Adipor2, and T‐cadherin protein levels were determined using PAGE‐SDS and western blot. Densitometric signal normalized to Ponceau Red. Data presented as boxplots; groups compared using Student's *t*‐tests (NS, *p* = 0.033, *p* = 0.039, respectively, as indicated). Representative blots are shown on the right. CTL group: *N* = 7, HLUI group: *N* = 5.

Here, we investigated ApN pathway (co)receptors expression at the mRNA and protein levels in HLUI‐induced disused muscles (Figure 5). Globally, the data indicate that the effect of muscle disuse differs in slow‐twitch *Soleus* (Figure 5a,b) and fast‐twitch *TA* muscles (Figure 5c,d). Indeed, HLUI in mouse *Soleus* was associated with a decrease in *Adipor1* (encoding Adipor1), *Adipor2* (encoding Adipor2), and *Cdh13* (encoding T‐cadherin) mRNA levels as compared to CTL (*p* < 0.01, Student's *t*‐test) (Figure 5a). At the protein level, the decreased Adipor1 protein level was not statistically significant in the *Soleus* of HLUI mice (24.25 ± 23.32) as compared to CTL (38.65 ± 11.42) (Student's *t*‐test), and we observed relatively high variability in the HLUI group (Figure S6). *Adipor2* expression decline is consistent with the significant decrease of Adipor2 protein level (30.11 ± 7.13) as compared to CTL mice (49.30 ± 13.50) (*p* < 0.05, Student's *t*‐test) (Figure 5b, Figure S7). As concerns the co‐receptor T‐cadherin, the immunodetection on western blot allowed us to detect two bands corresponding to T‐cadherin with (130 kDa) and without (100 kDa) its prodomain, as described in (Fukuda et al., 2017; Tanaka et al., 2019). Densitometric analysis indicated that the total amount of T‐cadherin was increased in disused *Soleus* muscles (28.50 ± 9.51) as compared to CTL (18.34 ± 5.33) (*p* < 0.05, Student's *t*‐test) (Figure 5b). This difference was observed for the 100 kDa form (*p* < 0.05, Student's *t*‐test) and was at the limit of statistical significance for the 130 kDa form (*p* = 0.057, Student's *t*‐test) (Figure S8).

Contrary to data obtained in the *Soleus* muscle, HLUI in mouse *TA* is not associated with modifications of ApN (co)receptor expression, neither at the mRNA, nor at protein levels (Figure 5c,d and Figures [Link], [Link] and S9).

## 4 DISCUSSION

Hindlimb Unloading (HLU) models are widely used to mimic muscle disuse in rodents (Bodine, 2013; Marzuca‐Nassr et al., 2019; Morey‐Holton et al., 2005). Here, this model was coupled with hindlimb immobilization and optimized to reduce mouse stress (acclimatization period, social interactions, displacement with forelimbs, …), thus permitting minimization of bias in assessing disuse‐mediated muscle effects. These optimizations successfully limited body weight (b.w.) loss in HLUI mice compared to other studies in HLU rodent models (Moustafa, 2021; Tousen et al., 2020). The b.w. is an important parameter to control in HLUI experiments to avoid confounding factors. Indeed, b.w. loss is known to be associated with an activation of muscle proteolysis pathways (Kangalgil et al., 2024; Kvedaras et al., 2020) and to modify ApN pathway components.

### 4.1 HLUI induces a DMA whose severity depends on fiber type, muscle function, and positioning

Numerous studies reported that fast and slow fiber types are not equivalent in terms of susceptibility to atrophy, the primarily affected subtype depending on the atrophic condition. Type I fibers are reported to be more sensitive to inactivity and microgravity, whereas type II fibers seem more vulnerable to cachexia, diabetes, and aging (sarcopenia) (Ciciliot et al., 2013; Wang & Pessin, 2013). Moreover, it is also generally accepted that muscle physiological function and position constitute determinant factors that may promote or limit atrophy development (Ciciliot et al., 2013; Wang & Pessin, 2013). In our adapted model, HLUI had only a moderate effect in the slow‐twitch *Soleus* muscle, whereas the *TA* muscle exhibited a more severe DMA. This result might be surprising since muscles having a high proportion of type I myofibers are reported to be more sensitive to unloading than predominantly fast‐type muscles (Oliveira et al., 2019; Thomason & Booth, 1990). Our results could be explained by the positioning of the *Soleus* muscle during immobilization. Indeed, in our experimental condition, mouse ankles are in a neutral position, close to the dorsiflexed position, the plantarflexion being commonly considered “unnatural”. Ankle joint immobilization in the dorsiflexal position stretches the *Soleus* muscle, a condition associated with less severe atrophy than shortened muscles, due to the anabolic effect of muscle stretching itself (Warneke et al., 2023) but also to persistent isometric contractions (Baker & Matsumoto, 1988; Fujita et al., 2009; Goldspink, 1977). Conversely, ankle joint immobilization in HLUI mice shortened the *TA* muscle, a position known to maximize atrophy development (Fujita et al., 2009). Muscle positioning during immobilization could also explain the greater impact of HLUI on IIa and IIb myofibers, one mechanistic hypothesis being a reduced protection against atrophy through PGC1α and NFAT signaling pathways, compared to type I fibers (Wang & Pessin, 2013).

### 4.2 HLUI induces a slow‐to‐fast myofiber shift whose kinetic is muscle‐type dependent

A slow‐to‐fast myofiber switch has been largely described in the context of muscle disuse (Baehr et al., 2022; Bodine, 2013; Shenkman, 2016; Wang et al., 2008), and a 14‐day HLU is reported to provoke this transition in the antigravitational *Soleus* muscle (Bodine, 2013; Marzuca‐Nassr et al., 2017; Wang et al., 2017). In the adapted HLUI model, myofiber‐type proportions were unchanged in the *Soleus* muscle of HLUI mice. However, we observed an increased proportion of type I fibers that are also positive for the type IIa marker. Indeed, as introduced by Pette and Staron (Pette & Staron, 2000, 1993; Zhang et al., 2010), if myofibers are commonly classified into major subtypes, they form a highly dynamic continuum where MyHC expression can adapt notably in response to functional demands. Since hybrid I/IIa myofibers are known to be implicated in fiber‐type transitioning (Blottner et al., 2020; Medler, 2019), their increased number suggests an ongoing switch from type I to type IIa myofibers in the HLUI *Soleus* muscle. However, persistent isometric contraction in the *Soleus* muscle could contribute to delay myofiber switch upon HLUI, this muscle being likely in a transitioning state. On the other hand, HLUI *TA* muscles present evidence of a shift towards a faster phenotype. Indeed, most type I myofibers not only express the type IIa marker but also have a significantly reduced proportion.

### 4.3 HLUI enhances ApN plasma level and disturbs oligomeric form proportions

Given the myoprotective effects of ApN (Abou‐Samra, Selvais, Boursereau, et al., 2020; Abou‐Samra, Selvais, Dubuisson, & Brichard, 2020; Iwabu et al., 2010; Jiang et al., 2019), we investigated whether DMA is accompanied by alterations in its mRNA expression in disused muscles, plasmatic level, circulating form proportion, and muscular receptor expression.

At a systemic level, we observed increased levels of ApN in the plasma of HLUI mice, as well as disturbances of LMW and MMW circulating form proportions. Increased ApN plasma levels were suggested to be implicated in sarcopenia‐mediated muscle wasting, as higher ApN plasma levels are associated with low skeletal muscle mass and higher mortality (Baker et al., 2019; Menzaghi & Trischitta, 2018). However, this hypothesis was rejected by C. Walowski et al., who suggested that muscle mass loss is not causally related to ApN in older adults. The authors attribute “the ApN paradox” to an age‐related decrease of Insulin‐like Growth Factor (IGF‐1), negatively associated with ApN plasma level (Walowski et al., 2023). Conversely, in a murine model of muscle dystrophy (*mdx* mice, a model of Duchenne Muscular Dystrophy (DMD)), lower ApN plasma levels were reported (Abou‐Samra et al., 2015). Here, several factors may contribute to the increased ApN plasma levels. *Adipoq* mRNA level is not significantly modified in the disused muscles, suggesting a modification of its secretion by other secretory organs, such as adipose tissue. Potential modifications in *Adipoq* post‐transcriptional processes in HLUI mouse muscles cannot be excluded. Since ApN circulating forms do not interconvert in plasma (Peake et al., 2005), the observed decrease in LMW proportion in favor of MMW in HLUI plasma is probably the result of variations in ApN post‐translational modifications upon disuse. Even if the HMW ApN form is commonly considered the most active, the consequences of disturbed MMW and LMW form proportions have, to date, not been elucidated, particularly in skeletal muscle. Moreover, even if gApN and full‐length ApN have been reported to exhibit different binding strengths for ADIPOR1 and ADIPOR2 (Kadowaki et al., 2006), little is known about the respective affinities of MMW and LMW forms for those receptors.

### 4.4 HLUI induces muscle‐type dependent alterations of Adiporeceptors that are not associated to DMA severity

Even if ApN plasma level increases upon disuse, our results suggest an ApN resistance in the *Soleus* muscle. Indeed, *Adipor1* and *Adipor2* expressions are downregulated in the HLUI *Soleus* muscle as compared to controls. Accordingly, Goto et al. observed a similar decline in *Adipor1* but not in *Adipor2* mRNA levels in the *Soleus* muscle after 14 days of HLU without immobilization (Goto et al., 2013). Moreover, they demonstrated that functional overloading and muscle regrowth following HLU are accompanied by an upregulation of *Adipor1* mRNA levels, thus highlighting a link between mechanical loading and ApN pathway regulation (Goto et al., 2013). On the other hand, *Adipor1* and *Adipor2* mRNA down‐regulation may also result from a disuse‐mediated hyperinsulinemia (Hamburg et al., 2007; Hirose et al., 2000; Kakehi et al., 2021; Mondon et al., 1992), as *Adipor1* and *Adipor2* mRNA levels were reported to be inversely regulated by insulin in physiological conditions, via Phosphoinositide 3‐kinase/Foxo1 dependent pathways (Tsuchida et al., 2004). However, *Adipor1* and *Adipor2*, expressions were found unchanged in HLUI *TA* muscles. Although hyperinsulinemia was not directly assessed in our study, we can reasonably assume that if present, such hyperinsulinemia would have affected similarly *Adipor1* and *Adipor2* mRNA levels in the *TA* muscle.

Moreover, the HLUI‐mediated adiporeceptor down‐regulation is associated with decreased Adipor2 protein levels in the *Soleus* muscle. Adipor1 protein level also tends to decrease with HLUI, but we notice a higher inter‐individual variability. Such alterations in Adipor protein level can prevent ApN biological activities. Indeed, the Adipor1/AMPK/SIRT1/PGC1α axis (Yamauchi et al., 2007) was reported as essential for the maintenance of type I myofibers and oxidative metabolism (Iwabu et al., 2010). Consequently, this suggests that the decreased adiporeceptor protein level is involved in the slow‐to‐fast myofiber switch occurring in the *Soleus* muscle of HLUI mice. Furthermore, muscle disuse is known to mediate a PGC1α hypoactivation (Cannavino et al., 2014; Feng et al., 2016) that may limit its repressive action on FoxO3, a transcription factor that promotes the expression of the MAFbx Ubiquitin Ligases (Atrogin 1) implicated in myofiber atrophy (Bodine, 2013; Ciciliot et al., 2013; Sandri et al., 2004). The consequences of a downregulation of Adipor2 are unclear, as this receptor has been poorly studied in skeletal muscle due to its reduced abundance in this tissue compared to Adipor1. However, Adipor2 activation by ApN is known to activate the expression of PPARs ligands, thus stimulating glucose and lipid homeostasis (Yamauchi et al., 2014, 2003; Yamauchi & Kadowaki, 2013). Adipor2 protein level reduction in HLUI *Soleus* muscles could thus have consequences at the metabolic level. Interestingly, despite *Cdh13* mRNA decrease by disuse, T‐cadherin protein level appears increased in the *Soleus* muscle. Such an increase in T‐cadherin protein level is consistent with the elevated ApN plasma level, as T‐cadherin protein, but not *Cdh13* mRNA, is known to be increased by ApN in endothelial cells *in vivo* and *in vitro* via the suppression of an endogenous GPI phospholipase D implicated in T‐cadherin cleavage (Matsuda et al., 2015). It is now well established that T‐cadherin acts locally to maintain ApN at the myofiber membrane. However, T‐cadherin has no transmembrane domain and is then unable to activate intracellular pathways by itself (Fukuda et al., 2017; Tanaka et al., 2019).

Contrary to the *Soleus*, Adipor1, Adipor2, and T‐cadherin protein levels appear unaffected by disuse in the *TA* muscle. Such results are consistent with the absence of modifications in ApN (co)receptor expression in this muscle. Disuse‐mediated ApN pathway alterations are thus rather muscle‐type dependent than related to the severity of muscle atrophy. Our data therefore highlight the necessity to better understand fiber‐type specific alterations of adiporeceptors in muscle disuse. In prospect of our study, potential change in adiporeceptors' membrane location, internalization rate, and recycling (Buechler et al., 2010; Ding et al., 2009), as well as the presence of Adipor homo‐ and heterodimers (Almabouada et al., 2013; Kosel et al., 2010), have also to be better deciphered in this pathological context as factors influencing ApN effects (Almabouada et al., 2013; Buechler et al., 2010; Ding et al., 2009; Kosel et al., 2010).

## 5 CONCLUSIONS

In conclusion, HLUI in mice induces a fiber‐type‐dependent atrophy accompanied by a type I/IIa myofiber switch. Muscle activity in physiological conditions (anti‐gravity vs. running) and positioning during immobilization (stretched vs. shortened) constitute additional factors influencing the kinetics of disuse consequences. Muscle alterations occur concomitantly with an elevation of ApN plasma level and disturbances in oligomeric form proportion. Disuse‐mediated adiporeceptor alterations occur in a muscle‐type‐dependent manner rather than being related to the severity of DMA. Further gain and loss‐of‐function studies are now needed to determine whether the ApN pathway changes upon disuse participate in a vicious cycle reinforcing muscle dysfunction in this pathological context.

## AUTHOR CONTRIBUTIONS

A.T., A.L., A.E.D., and S.S. conceived and designed the research; S.S., M.L., and V.J. performed the experiments; S.S. analyzed data; A.T. and S.S. interpreted results of experiments; A.T. and S.S. prepared figures; A.T. and S.S. drafted the manuscript; A.T., S.S., A.L., A.E.D., M.L., and V.J. edited and revised the manuscript; A.T., S.S., A.L., A.E.D., M.L., and V.J. approved the final version of the manuscript.

## FUNDING INFORMATION

S.S., and M.L., hold a FRIA doctoral fellowship (FC 41735/FC 47057) from the National Fund for Scientific Research (F.R.S‐F.N.R.S), Belgium. The authors acknowledge funding from FRMH (Fonds pour la Recherche Médicale dans le Hainaut (FRMH), Grant call 2015) and ABMM (Association Belge contre les Maladies Neuro‐Musculaires, Grant call 2015).

## CONFLICT OF INTEREST STATEMENT

The authors declare they have no conflict of interest, financial or otherwise.

## Supporting information


**Table S1.** Primers used in RTqPCR analyses.

**Figure S1.** Food consumption in CTL and HLUI mice. (A) Food consumption was measured daily. Data plotted as mean ± SD and compared using a Two‐way ANOVA repeated measures (*p* < 0.001, HLUI vs. CTL). (B) Mean daily food consumption. Data presented as boxplots; groups compared using a Student’s *t*‐test (NS). CTL group: *N* = 10, HLUI group: *N* = 11.

**Figure S2.** Early effects of HLUI (day 3) on mouse bodyweight and *Fbxo 32* expression in *Soleus* and *Tibialis anterior* muscles. (A) Daily measurements normalized to b.w. at D0 (defined as the 100% baseline). Data presented as mean ± SD and groups compared using a Two‐way ANOVA repeated measures (*p* = 0.034, *p* = 0.022, as indicated). (B) *Fbxo32* mRNA level was assessed in (C) the *Soleus* and (D) the *Tibialis anterior* muscles by RTqPCR. Data presented as boxplot; groups compared using a Student’s *t*‐test (*p* = 0.001, *p* = 0.007, as indicated). CTL group: *N* = 6, HLUI group: *N* = 6.

**Figure S3.** Effects of HLUI on type IIb and unstained (IIx) myofibers in the *Soleus* muscle. (A) Representative fields. (B–C) Cross‐sectional Area (CSA), myofiber CSA distribution, and Minimum Feret’s Diameter (MFD) in type IIb (B) and unstained (C) myofibers. Data presented as in Fig.2. CSA: Student’s *t*‐test (NS). Myofiber CSA distribution: Statistical tests were not performed because of the scarcity of type IIb fibers in the *Soleus* muscle. For unstained myofibers, groups were compared using a Chi‐square (Chi^2^) test (NS). MFD: Student’s *t*‐test (*p* = 0.026, as indicated). CTL group: *N* = 5, HLUI group: *N* = 5.

**Figure S4.** Effects of HLUI on type I and unstained (IIx) myofibers in the *Tibialis anterior* muscle. (A) Representative fields. (B‐C) Cross‐sectional Area (CSA), myofiber CSA distribution, and Minimum Feret’s Diameter (MFD) in type I (B) and unstained myofibers (C). Data presented as in Fig.2. CSA: Student’s *t*‐test in type I myofibers; Welch’s *t*‐test in unstained myofibers (NS). Myofiber CSA distribution: Chi‐square (Chi^2^) tests (*p*<0.001, as indicated). MFD: Student’s *t*‐test in type I myofibers, Welch’s *t*‐test in unstained myofibers (NS). CTL group: *N* = 5, HLUI group: *N* = 5.

**Figure S5.** Adiponectin circulating forms: western blot immunodetection and corresponding Ponceau Red.

**Figure S6.** Adipor1: western blot immunodetection and corresponding Ponceau Red in (A) *Soleus* and (B) *Tibialis anterior* muscles.

**Figure S7.** Adipor2: western blot immunodetection and corresponding Ponceau Red in (A) *Soleus* and (B) *Tibialis anterior* muscles.

**Figure S8.** Effect of HLUI on mature (100kDa) and pro‐domain bearing (130kDa) T‐cadherin protein levels in the *Soleus* muscle. (A) T‐cadherin protein levels were determined using PAGE‐SDS and western blot. Densitometric signal normalized to Ponceau Red. Data presented as boxplots; groups compared using Student’s *t*‐tests (*p* = 0,039, as indicated). CTL group: *N* = 7, HLUI group: *N* = 5. (B) Representative blots.

**Figure S9.** Effect of HLUI on mature (100kDa) and pro‐domain bearing (130kDa) T‐cadherin protein level in the *Tibialis anterior muscle*. (A) T‐cadherin protein levels were determined using PAGE‐SDS and western blot. Densitometric signal normalized to Ponceau Red. Data presented as boxplot; groups compared using Student’s *t*‐tests (NS). CTL group: *N* = 7, HLUI group: *N* = 5. (B) Representative blots.

## Data Availability

All data supporting the findings of this study are available within the article and its Supporting Information.

## References

[phy270602-bib-0001] Abou‐Samra, M. , Lecompte, S. , Schakman, O. , Noel, L. , Many, M. C. , Gailly, P. , & Brichard, S. M. (2015). Involvement of adiponectin in the pathogenesis of dystrophinopathy. Skeletal Muscle, 5, 25. 10.1186/s13395-015-0051-9 26257862 PMC4528853

[phy270602-bib-0002] Abou‐Samra, M. , Selvais, C. M. , Boursereau, R. , Lecompte, S. , Noel, L. , & Brichard, S. M. (2020). AdipoRon, a new therapeutic prospect for Duchenne muscular dystrophy. Journal of Cachexia, Sarcopenia and Muscle, 11, 518–533. 10.1002/jcsm.12531 31965757 PMC7113498

[phy270602-bib-0003] Abou‐Samra, M. , Selvais, C. M. , Dubuisson, N. , & Brichard, S. M. (2020). Adiponectin and its mimics on skeletal muscle: Insulin sensitizers, fat burners, exercise mimickers, muscling pills … or everything together? International Journal of Molecular Sciences, 21, 2620. 10.3390/ijms21072620 32283840 PMC7178193

[phy270602-bib-0004] Almabouada, F. , Diaz‐Ruiz, A. , Rabanal‐Ruiz, Y. , Peinado, J. R. , Vazquez‐Martinez, R. , & Malagon, M. M. (2013). Adiponectin receptors form homomers and Heteromers exhibiting distinct ligand binding and intracellular signaling properties. Journal of Biological Chemistry, 288, 3112–3125. 10.1074/jbc.M112.404624 23255609 PMC3561534

[phy270602-bib-0005] Amin, R. H. , Mathews, S. T. , Camp, H. S. , Ding, L. , & Leff, T. (2010). Selective activation of PPARγ in skeletal muscle induces endogenous production of adiponectin and protects mice from diet‐induced insulin resistance. American Journal of Physiology. Endocrinology and Metabolism, 298, E28–E37. 10.1152/ajpendo.00446.2009 19843873

[phy270602-bib-0006] Arc‐Chagnaud, C. , Py, G. , Fovet, T. , Roumanille, R. , Demangel, R. , Pagano, A. F. , Delobel, P. , Blanc, S. , Jasmin, B. J. , Blottner, D. , Salanova, M. , Gomez‐Cabrera, M.‐C. , Viña, J. , Brioche, T. , & Chopard, A. (2020). Evaluation of an antioxidant and anti‐inflammatory cocktail against human hypoactivity‐induced skeletal muscle deconditioning. Frontiers in Physiology, 11, 71. 10.3389/fphys.2020.00071 32116779 PMC7028694

[phy270602-bib-0007] Atherton, P. J. , Greenhaff, P. L. , Phillips, S. M. , Bodine, S. C. , Adams, C. M. , & Lang, C. H. (2016). Control of skeletal muscle atrophy in response to disuse: Clinical/preclinical contentions and fallacies of evidence. American Journal of Physiology. Endocrinology and Metabolism, 311, E594–E604. 10.1152/ajpendo.00257.2016 27382036 PMC5142005

[phy270602-bib-0008] Baehr, L. M. , Hughes, D. C. , Waddell, D. S. , & Bodine, S. C. (2022). SnapShot: Skeletal muscle atrophy. Cell, 185, 1618–1618.e1. 10.1016/j.cell.2022.03.028 35487192

[phy270602-bib-0009] Baker, J. F. , Newman, A. B. , Kanaya, A. , Leonard, M. B. , Zemel, B. , Miljkovic, I. , Long, J. , Weber, D. , & Harris, T. B. (2019). The adiponectin paradox in the elderly: Associations with body composition, physical functioning, and mortality. The Journals of Gerontology: Series A, 74, 247–253. 10.1093/gerona/gly017 PMC6333931 29438496

[phy270602-bib-0010] Baker, J. H. , & Matsumoto, D. E. (1988). Adaptation of skeletal muscle to immobilization in a shortened position. Muscle and Nerve, 11, 231–244. 10.1002/mus.880110308 3352658

[phy270602-bib-0011] Balasubramanian, P. , Schaar, A. E. , Gustafson, G. E. , Smith, A. B. , Howell, P. R. , Greenman, A. , Baum, S. , Colman, R. J. , Lamming, D. W. , Diffee, G. M. , & Anderson, R. M. (2022). Adiponectin receptor agonist AdipoRon improves skeletal muscle function in aged mice. eLife, 11, e71282. 10.7554/eLife.71282 35297761 PMC8963882

[phy270602-bib-0012] Baldwin, K. M. , Haddad, F. , Pandorf, C. E. , Roy, R. R. , & Edgerton, V. R. (2013). Alterations in muscle mass and contractile phenotype in response to unloading models: Role of transcriptional/pretranslational mechanisms. Frontiers in Physiology, 4, 284. 10.3389/fphys.2013.00284 24130531 PMC3795307

[phy270602-bib-0013] Basu, R. , Pajvani, U. B. , Rizza, R. A. , & Scherer, P. E. (2007). Selective downregulation of the high molecular weight form of adiponectin in hyperinsulinemia and in type 2 diabetes: Differential regulation from nondiabetic subjects. Diabetes, 56, 2174–2177. 10.2337/db07-0185 17513700

[phy270602-bib-0014] Bloemberg, D. , & Quadrilatero, J. (2012). Rapid determination of myosin heavy chain expression in rat, mouse, and human skeletal muscle using multicolor immunofluorescence analysis. PLoS One, 7, e35273. 10.1371/journal.pone.0035273 22530000 PMC3329435

[phy270602-bib-0015] Blottner, D. , Hastermann, M. , Weber, R. , Lenz, R. , Gambara, G. , Limper, U. , Rittweger, J. , Bosutti, A. , Degens, H. , & Salanova, M. (2020). Reactive jumps preserve skeletal muscle structure, phenotype, and myofiber oxidative capacity in bed rest. Frontiers in Physiology, 10, 1527. 10.3389/fphys.2019.01527 32009969 PMC6974579

[phy270602-bib-0016] Bodine, S. C. (2013). Disuse‐induced muscle wasting. The International Journal of Biochemistry & Cell Biology, 45, 2200–2208. 10.1016/j.biocel.2013.06.011 23800384 PMC3856924

[phy270602-bib-0017] Bodine, S. C. , & Baehr, L. M. (2014). Skeletal muscle atrophy and the E3 ubiquitin ligases MuRF1 and MAFbx/atrogin‐1. American Journal of Physiology. Endocrinology and Metabolism, 307, E469–E484. 10.1152/ajpendo.00204.2014 25096180 PMC4166716

[phy270602-bib-0018] Bodine, S. C. , Latres, E. , Baumhueter, S. , Lai, V. K.‐M. , Nunez, L. , Clarke, B. A. , Poueymirou, W. T. , Panaro, F. J. , Na, E. , Dharmarajan, K. , Pan, Z.‐Q. , Valenzuela, D. M. , DeChiara, T. M. , Stitt, T. N. , Yancopoulos, G. D. , & Glass, D. J. (2001). Identification of ubiquitin ligases required for skeletal muscle atrophy. Science, 294, 1704–1708. 10.1126/science.1065874 11679633

[phy270602-bib-0019] Boelens, Y. F. N. , Melchers, M. , & van Zanten, A. R. H. (2022). Poor physical recovery after critical illness: Incidence, features, risk factors, pathophysiology, and evidence‐based therapies. Current Opinion in Critical Care, 28, 409–416. 10.1097/MCC.0000000000000955 35796071 PMC9594146

[phy270602-bib-0020] Briguet, A. , Courdier‐Fruh, I. , Foster, M. , Meier, T. , & Magyar, J. P. (2004). Histological parameters for the quantitative assessment of muscular dystrophy in the mdx‐mouse. Neuromuscular Disorders, 14, 675–682. 10.1016/j.nmd.2004.06.008 15351425

[phy270602-bib-0021] Buechler, C. , Wanninger, J. , & Neumeier, M. (2010). Adiponectin receptor binding proteins—Recent advances in elucidating adiponectin signalling pathways. FEBS Letters, 584, 4280–4286. 10.1016/j.febslet.2010.09.035 20875820

[phy270602-bib-0022] Cannavino, J. , Brocca, L. , Sandri, M. , Bottinelli, R. , & Pellegrino, M. A. (2014). PGC1‐α over‐expression prevents metabolic alterations and soleus muscle atrophy in hindlimb unloaded mice. The Journal of Physiology, 592, 4575–4589. 10.1113/jphysiol.2014.275545 25128574 PMC4287741

[phy270602-bib-0023] Ciciliot, S. , Rossi, A. C. , Dyar, K. A. , Blaauw, B. , & Schiaffino, S. (2013). Muscle type and fiber type specificity in muscle wasting. The International Journal of Biochemistry & Cell Biology, 45, 2191–2199. 10.1016/j.biocel.2013.05.016 23702032

[phy270602-bib-0024] Cuthbertson, B. H. , Roughton, S. , Jenkinson, D. , MacLennan, G. , & Vale, L. (2010). Quality of life in the five years after intensive care: A cohort study. Critical Care, 14, R6. 10.1186/cc8848 20089197 PMC2875518

[phy270602-bib-0025] Denzel, M. S. , Scimia, M.‐C. , Zumstein, P. M. , Walsh, K. , Ruiz‐Lozano, P. , & Ranscht, B. (2010). T‐cadherin is critical for adiponectin‐mediated cardioprotection in mice. The Journal of Clinical Investigation, 120, 4342–4352. 10.1172/JCI43464 21041950 PMC2993592

[phy270602-bib-0026] Ding, Q. , Wang, Z. , & Chen, Y. (2009). Endocytosis of adiponectin receptor 1 through a clathrin‐ and Rab5‐dependent pathway. Cell Research, 19, 317–327. 10.1038/cr.2008.299 18982021

[phy270602-bib-0027] Dos Santos, C. , Hussain, S. N. A. , Mathur, S. , Picard, M. , Herridge, M. , Correa, J. , Bain, A. , Guo, Y. , Advani, A. , Advani, S. L. , Tomlinson, G. , Katzberg, H. , Streutker, C. J. , Cameron, J. I. , Schols, A. , Gosker, H. R. , & Batt, J. (2016). Mechanisms of chronic muscle wasting and dysfunction after an intensive care unit stay. A pilot study. American Journal of Respiratory and Critical Care Medicine, 194, 821–830. 10.1164/rccm.201512-2344OC 27058306

[phy270602-bib-0028] Du, F. , Wang, J. , Gao, Y. , Wang, H. , Wang, Q. , Jiang, S. , & Goswami, N. (2011). A hind limb disuse model inducing extensor digitorum longus atrophy in rats: Tail suspension‐immobilization. Aviation, Space, and Environmental Medicine, 82, 689–693. 10.3357/asem.2984.2011 21748906

[phy270602-bib-0029] Feng, H.‐Z. , Chen, X. , Malek, M. H. , & Jin, J.‐P. (2016). Slow recovery of the impaired fatigue resistance in postunloading mouse soleus muscle corresponding to decreased mitochondrial function and a compensatory increase in type I slow fibers. American Journal of Physiology‐Cell Physiology, 310, C27–C40. 10.1152/ajpcell.00173.2015 26447205 PMC4698445

[phy270602-bib-0030] Fiaschi, T. , Cirelli, D. , Comito, G. , Gelmini, S. , Ramponi, G. , Serio, M. , & Chiarugi, P. (2009). Globular adiponectin induces differentiation and fusion of skeletal muscle cells. Cell Research, 19, 584–597. 10.1038/cr.2009.39 19350052

[phy270602-bib-0031] Fiaschi, T. , Giannoni, E. , Taddei, M. L. , & Chiarugi, P. (2012). Globular adiponectin activates motility and regenerative traits of muscle satellite cells. PLoS One, 7, e34782. 10.1371/journal.pone.0034782 22629295 PMC3356356

[phy270602-bib-0032] Fiaschi, T. , Magherini, F. , Gamberi, T. , Modesti, P. A. , & Modesti, A. (2014). Adiponectin as a tissue regenerating hormone: More than a metabolic function. Cellular and Molecular Life Sciences, 71, 1917–1925. 10.1007/s00018-013-1537-4 24322911 PMC11113778

[phy270602-bib-0033] Fujita, N. , Fujimoto, T. , Tasaki, H. , Arakawa, T. , Matsubara, T. , & Miki, A. (2009). Influence of muscle length on muscle atrophy in the mouse tibialis anterior and soleus muscles. Biomedical Research, 30, 39–45. 10.2220/biomedres.30.39 19265262

[phy270602-bib-0034] Fukuda, S. , Kita, S. , Obata, Y. , Fujishima, Y. , Nagao, H. , Masuda, S. , Tanaka, Y. , Nishizawa, H. , Funahashi, T. , Takagi, J. , Maeda, N. , & Shimomura, I. (2017). The unique prodomain of T‐cadherin plays a key role in adiponectin binding with the essential extracellular cadherin repeats 1 and 2. Journal of Biological Chemistry, 292, 7840–7849. 10.1074/jbc.M117.780734 28325833 PMC5427265

[phy270602-bib-0035] Gamberi, T. , Modesti, A. , Magherini, F. , D'Souza, D. M. , Hawke, T. , & Fiaschi, T. (2016). Activation of autophagy by globular adiponectin is required for muscle differentiation. Biochimica et Biophysica Acta, Molecular Cell Research, 1863, 694–702. 10.1016/j.bbamcr.2016.01.016 26826036

[phy270602-bib-0036] Gao, Y. , Arfat, Y. , Wang, H. , & Goswami, N. (2018). Muscle atrophy induced by mechanical unloading: Mechanisms and potential countermeasures. Frontiers in Physiology, 9, 235. 10.3389/fphys.2018.00235 29615929 PMC5869217

[phy270602-bib-0037] Goldspink, D. F. (1977). The influence of immobilization and stretch on protein turnover of rat skeletal muscle. The Journal of Physiology, 264, 267–282. 10.1113/jphysiol.1977.sp011667 839454 PMC1307757

[phy270602-bib-0038] Goto, A. , Ohno, Y. , Ikuta, A. , Suzuki, M. , Ohira, T. , Egawa, T. , Sugiura, T. , Yoshioka, T. , Ohira, Y. , & Goto, K. (2013). Up‐regulation of adiponectin expression in antigravitational soleus muscle in response to unloading followed by reloading, and functional overloading in mice. PLoS One, 8, e81929. 10.1371/journal.pone.0081929 24324732 PMC3855747

[phy270602-bib-0039] Hamburg, N. M. , McMackin, C. J. , Huang, A. L. , Shenouda, S. M. , Widlansky, M. E. , Schulz, E. , Gokce, N. , Ruderman, N. B. , Keaney, J. F. , & Vita, J. A. (2007). Physical inactivity rapidly induces insulin resistance and microvascular dysfunction in healthy volunteers. Arteriosclerosis, Thrombosis, and Vascular Biology, 27, 2650–2656. 10.1161/ATVBAHA.107.153288 17932315 PMC2596308

[phy270602-bib-0040] Hara, K. , Horikoshi, M. , Yamauchi, T. , Yago, H. , Miyazaki, O. , Ebinuma, H. , Imai, Y. , Nagai, R. , & Kadowaki, T. (2006). Measurement of the high‐molecular weight form of adiponectin in plasma is useful for the prediction of insulin resistance and metabolic syndrome. Diabetes Care, 29, 1357–1362. 10.2337/dc05-1801 16732021

[phy270602-bib-0041] Hirose, M. , Kaneki, M. , Sugita, H. , Yasuhara, S. , & Martyn, J. A. J. (2000). Immobilization depresses insulin signaling in skeletal muscle. American Journal of Physiology. Endocrinology and Metabolism, 279, E1235–E1241. 10.1152/ajpendo.2000.279.6.E1235 11093909

[phy270602-bib-0042] Iwabu, M. , Yamauchi, T. , Okada‐Iwabu, M. , Sato, K. , Nakagawa, T. , Funata, M. , Yamaguchi, M. , Namiki, S. , Nakayama, R. , Tabata, M. , Ogata, H. , Kubota, N. , Takamoto, I. , Hayashi, Y. K. , Yamauchi, N. , Waki, H. , Fukayama, M. , Nishino, I. , Tokuyama, K. , … Kadowaki, T. (2010). Adiponectin and AdipoR1 regulate PGC‐1α and mitochondria by Ca2+ and AMPK/SIRT1. Nature, 464, 1313–1319. 10.1038/nature08991 20357764

[phy270602-bib-0043] Jiang, Q. , Cheng, X. , Cui, Y. , Xia, Q. , Yan, X. , Zhang, M. , Lan, G. , Liu, J. , Shan, T. , & Huang, Y. (2019). Resveratrol regulates skeletal muscle fibers switching through the AdipoR1‐AMPK‐PGC‐1α pathway. Food & Function, 10, 3334–3343. 10.1039/c8fo02518e 31095141

[phy270602-bib-0044] Kadowaki, T. , Yamauchi, T. , Kubota, N. , Hara, K. , Ueki, K. , & Tobe, K. (2006). Adiponectin and adiponectin receptors in insulin resistance, diabetes, and the metabolic syndrome. The Journal of Clinical Investigation, 116, 1784–1792. 10.1172/JCI29126 16823476 PMC1483172

[phy270602-bib-0045] Kakehi, S. , Tamura, Y. , Ikeda, S. , Kaga, N. , Taka, H. , Ueno, N. , Shiuchi, T. , Kubota, A. , Sakuraba, K. , Kawamori, R. , & Watada, H. (2021). Short‐term physical inactivity induces diacylglycerol accumulation and insulin resistance in muscle via lipin1 activation. American Journal of Physiology. Endocrinology and Metabolism, 321, E766–E781. 10.1152/ajpendo.00254.2020 34719943

[phy270602-bib-0046] Kangalgil, M. , Küçük, A. O. , Ulusoy, H. , & Özçelik, A. Ö. (2024). Nutrition determinants of acute skeletal muscle loss in critically ill patients: A prospective observational cohort study. Nutrition in Clinical Practice, 39, 579–588. 10.1002/ncp.11086 37877164

[phy270602-bib-0047] Kosel, D. , Heiker, J. T. , Juhl, C. , Wottawah, C. M. , Blüher, M. , Mörl, K. , & Beck‐Sickinger, A. G. (2010). Dimerization of adiponectin receptor 1 is inhibited by adiponectin. Journal of Cell Science, 123, 1320–1328. 10.1242/jcs.057919 20332107

[phy270602-bib-0048] Krause, M. , Milne, K. , & Hawke, T. (2019). Adiponectin—Consideration for its role in skeletal muscle health. International Journal of Molecular Sciences, 20, 1528. 10.3390/ijms20071528 30934678 PMC6480271

[phy270602-bib-0049] Krause, M. P. , Liu, Y. , Vu, V. , Chan, L. , Xu, A. , Riddell, M. C. , Sweeney, G. , & Hawke, T. J. (2008). Adiponectin is expressed by skeletal muscle fibers and influences muscle phenotype and function. American Journal of Physiology‐Cell Physiology, 295, C203–C212. 10.1152/ajpcell.00030.2008 18463233 PMC2493546

[phy270602-bib-0050] Kvedaras, M. , Minderis, P. , Krusnauskas, R. , & Ratkevicius, A. (2020). Effects of ten‐week 30% caloric restriction on metabolic health and skeletal muscles of adult and old C57BL/6J mice. Mechanisms of Ageing and Development, 190, 111320. 10.1016/j.mad.2020.111320 32735895

[phy270602-bib-0051] Liu, Y. , Palanivel, R. , Rai, E. , Park, M. , Gabor, T. V. , Scheid, M. P. , Xu, A. , & Sweeney, G. (2015). Adiponectin stimulates autophagy and reduces oxidative stress to enhance insulin sensitivity during high‐fat diet feeding in mice. Diabetes, 64, 36–48. 10.2337/db14-0267 25071026

[phy270602-bib-0052] Marzuca‐Nassr, G. N. , Murata, G. M. , Martins, A. R. , Vitzel, K. F. , Crisma, A. R. , Torres, R. P. , Mancini‐Filho, J. , Kang, J. X. , & Curi, R. (2017). Balanced diet‐fed Fat‐1 transgenic mice exhibit lower hindlimb suspension‐induced soleus muscle atrophy. Nutrients, 9, 1100. 10.3390/nu9101100 28984836 PMC5691716

[phy270602-bib-0053] Marzuca‐Nassr, G. N. , Vitzel, K. F. , Murata, G. M. , et al. (2019). Experimental model of HindLimb suspension‐induced skeletal muscle atrophy in rodents. In Methods in Molecular Biology (Vol. 1916, pp. 167–176). Springer. 10.1007/978-1-4939-8994-2_16 30535694

[phy270602-bib-0054] Matsuba, Y. , Goto, K. , Morioka, S. , Naito, T. , Akema, T. , Hashimoto, N. , Sugiura, T. , Ohira, Y. , Beppu, M. , & Yoshioka, T. (2009). Gravitational unloading inhibits the regenerative potential of atrophied soleus muscle in mice. Acta Physiologica, 196, 329–339. 10.1111/j.1748-1716.2008.01943.x 19040712

[phy270602-bib-0055] Matsuda, K. , Fujishima, Y. , Maeda, N. , Mori, T. , Hirata, A. , Sekimoto, R. , Tsushima, Y. , Masuda, S. , Yamaoka, M. , Inoue, K. , Nishizawa, H. , Kita, S. , Ranscht, B. , Funahashi, T. , & Shimomura, I. (2015). Positive feedback regulation between adiponectin and T‐cadherin impacts adiponectin levels in tissue and plasma of male mice. Endocrinology, 156, 934–946. 10.1210/en.2014-1618 25514086 PMC4330303

[phy270602-bib-0056] Medler, S. (2019). Mixing it up: The biological significance of hybrid skeletal muscle fibers. Journal of Experimental Biology, 222, jeb200832. 10.1242/jeb.200832 31784473

[phy270602-bib-0057] Menzaghi, C. , & Trischitta, V. (2018). The adiponectin paradox for all‐cause and cardiovascular mortality. Diabetes, 67, 12–22. 10.2337/dbi17-0016 29263167 PMC6181068

[phy270602-bib-0058] Mondon, C. E. , Rodnick, K. J. , Dolkas, C. B. , Azhar, S. , & Reaven, G. M. (1992). Alterations in glucose and protein metabolism in animals subjected to simulated microgravity. Advances in Space Research, 12, 169–177. 10.1016/0273-1177(92)90105-7 11537005

[phy270602-bib-0059] Morey‐Holton, E. , Globus, R. K. , Kaplansky, A. , & Durnova, G. (2005). The hindlimb unloading rat model: Literature overview, technique update and comparison with space flight data. In Advances in Space Biology and Medicine (Vol. 10, pp. 7–40). Elsevier. 16101103 10.1016/s1569-2574(05)10002-1

[phy270602-bib-0060] Moustafa, A. (2021). Hindlimb unloading‐induced reproductive suppression via downregulation of hypothalamic Kiss‐1 expression in adult male rats. Reproductive Biology and Endocrinology, 19, 37. 10.1186/s12958-021-00694-4 33663539 PMC7931529

[phy270602-bib-0061] Nunes, E. A. , Stokes, T. , McKendry, J. , Currier, B. S. , & Phillips, S. M. (2022). Disuse‐induced skeletal muscle atrophy in disease and nondisease states in humans: Mechanisms, prevention, and recovery strategies. American Journal of Physiology‐Cell Physiology, 322, C1068–C1084. 10.1152/ajpcell.00425.2021 35476500

[phy270602-bib-0062] Obata, Y. , Kita, S. , Koyama, Y. , Fukuda, S. , Takeda, H. , Takahashi, M. , Fujishima, Y. , Nagao, H. , Masuda, S. , Tanaka, Y. , Nakamura, Y. , Nishizawa, H. , Funahashi, T. , Ranscht, B. , Izumi, Y. , Bamba, T. , Fukusaki, E. , Hanayama, R. , Shimada, S. , … Shimomura, I. (2018). Adiponectin/T‐cadherin system enhances exosome biogenesis and decreases cellular ceramides by exosomal release. JCI Insight, 3, e99680. 10.1172/jci.insight.99680 29669945 PMC5931116

[phy270602-bib-0063] Oliveira, J. R. S. , Mohamed, J. S. , Myers, M. J. , Brooks, M. J. , & Alway, S. E. (2019). Effects of hindlimb suspension and reloading on gastrocnemius and soleus muscle mass and function in geriatric mice. Experimental Gerontology, 115, 19–31. 10.1016/j.exger.2018.11.011 30448397 PMC6366863

[phy270602-bib-0064] Pachitariu, M. , & Stringer, C. (2022). Cellpose 2.0: How to train your own model. Nature Methods, 19, 1634–1641. 10.1038/s41592-022-01663-4 36344832 PMC9718665

[phy270602-bib-0065] Pajvani, U. B. , Du, X. , Combs, T. P. , Berg, A. H. , Rajala, M. W. , Schulthess, T. , Engel, J. , Brownlee, M. , & Scherer, P. E. (2003). Structure‐function studies of the adipocyte‐secreted hormone Acrp30/adiponectin: IMPLICATIONS FOR METABOLIC REGULATION AND BIOACTIVITY. Journal of Biological Chemistry, 278, 9073–9085. 10.1074/jbc.M207198200 12496257

[phy270602-bib-0066] Pajvani, U. B. , Hawkins, M. , Combs, T. P. , Rajala, M. W. , Doebber, T. , Berger, J. P. , Wagner, J. A. , Wu, M. , Knopps, A. , Xiang, A. H. , Utzschneider, K. M. , Kahn, S. E. , Olefsky, J. M. , Buchanan, T. A. , & Scherer, P. E. (2004). Complex distribution, not absolute amount of adiponectin, correlates with thiazolidinedione‐mediated improvement in insulin sensitivity. Journal of Biological Chemistry, 279, 12152–12162. 10.1074/jbc.M311113200 14699128

[phy270602-bib-0067] Peake, P. W. , Kriketos, A. D. , Campbell, L. V. , Shen, Y. , & Charlesworth, J. A. (2005). The metabolism of isoforms of human adiponectin: Studies in human subjects and in experimental animals. European Journal of Endocrinology, 153, 409–417. 10.1530/eje.1.01978 16131604

[phy270602-bib-0068] Pette, D. , & Staron, R. (1993). The molecular diversity of mammalian muscle fibers. Physiology, 8, 153–157. 10.1152/physiologyonline.1993.8.4.153

[phy270602-bib-0069] Pette, D. , & Staron, R. S. (2000). Myosin isoforms, muscle fiber types, and transitions. Microscopy Research and Technique, 50, 500–509. 10.1002/1097-0029(20000915)50:6<500::AID-JEMT7>3.0.CO;2-7 10998639

[phy270602-bib-0070] Puthucheary, Z. , Harridge, S. , & Hart, N. (2010). Skeletal muscle dysfunction in critical care: Wasting, weakness, and rehabilitation strategies. Critical Care Medicine, 38, S676–S682. 10.1097/CCM.0b013e3181f2458d 21164414

[phy270602-bib-0071] Sandri, M. , Sandri, C. , Gilbert, A. , Skurk, C. , Calabria, E. , Picard, A. , Walsh, K. , Schiaffino, S. , Lecker, S. H. , & Goldberg, A. L. (2004). Foxo transcription factors induce the atrophy‐related ubiquitin ligase atrogin‐1 and cause skeletal muscle atrophy. Cell, 117, 399–412. 10.1016/s0092-8674(04)00400-3 15109499 PMC3619734

[phy270602-bib-0072] Shenkman, B. S. (2016). From slow to fast: Hypogravity‐induced remodeling of muscle fiber myosin phenotype. Acta Naturae, 8, 47–59. PMC5199206 28050266

[phy270602-bib-0073] Singh, A. K. , Shree, S. , Chattopadhyay, S. , Kumar, S. , Gurjar, A. , Kushwaha, S. , Kumar, H. , Trivedi, A. K. , Chattopadhyay, N. , Maurya, R. , Ramachandran, R. , & Sanyal, S. (2017). Small molecule adiponectin receptor agonist GTDF protects against skeletal muscle atrophy. Molecular and Cellular Endocrinology, 439, 273–285. 10.1016/j.mce.2016.09.013 27645900

[phy270602-bib-0074] Stringer, C. , Wang, T. , Michaelos, M. , & Pachitariu, M. (2021). Cellpose: A generalist algorithm for cellular segmentation. Nature Methods, 18, 100–106. 10.1038/s41592-020-01018-x 33318659

[phy270602-bib-0075] Tanaka, Y. , Kita, S. , Nishizawa, H. , Fukuda, S. , Fujishima, Y. , Obata, Y. , Nagao, H. , Masuda, S. , Nakamura, Y. , Shimizu, Y. , Mineo, R. , Natsukawa, T. , Funahashi, T. , Ranscht, B. , Fukada, S. , Maeda, N. , & Shimomura, I. (2019). Adiponectin promotes muscle regeneration through binding to T‐cadherin. Scientific Reports, 9, 16. 10.1038/s41598-018-37115-3 30626897 PMC6327035

[phy270602-bib-0076] Thomason, D. B. , & Booth, F. W. (1990). Atrophy of the soleus muscle by hindlimb unweighting. Journal of Applied Physiology, 68, 1–12. 10.1152/jappl.1990.68.1.1 2179205

[phy270602-bib-0077] Tian, L. , Luo, N. , Zhu, X. , Chung, B.‐H. , Garvey, W. T. , & Fu, Y. (2012). Adiponectin‐AdipoR1/2‐APPL1 signaling axis suppresses human foam cell formation: Differential ability of AdipoR1 and AdipoR2 to regulate inflammatory cytokine responses. Atherosclerosis, 221, 66–75. 10.1016/j.atherosclerosis.2011.12.014 22227293 PMC3288755

[phy270602-bib-0078] Tobias, I. S. , & Galpin, A. J. (2020). Moving human muscle physiology research forward: An evaluation of fiber type‐specific protein research methodologies. American Journal of Physiology‐Cell Physiology, 319, C858–C876. 10.1152/ajpcell.00107.2020 32783659

[phy270602-bib-0079] Tousen, Y. , Ichimaru, R. , Kondo, T. , Inada, M. , Miyaura, C. , & Ishimi, Y. (2020). The combination of soy isoflavones and resveratrol preserve bone mineral density in hindlimb‐unloaded mice. Nutrients, 12, 2043. 10.3390/nu12072043 32660008 PMC7400925

[phy270602-bib-0080] Tsuchida, A. , Yamauchi, T. , Ito, Y. , Hada, Y. , Maki, T. , Takekawa, S. , Kamon, J. , Kobayashi, M. , Suzuki, R. , Hara, K. , Kubota, N. , Terauchi, Y. , Froguel, P. , Nakae, J. , Kasuga, M. , Accili, D. , Tobe, K. , Ueki, K. , Nagai, R. , & Kadowaki, T. (2004). Insulin/Foxo1 pathway regulates expression levels of adiponectin receptors and adiponectin sensitivity. The Journal of Biological Chemistry, 279, 30817–30822. 10.1074/jbc.M402367200 15123605

[phy270602-bib-0081] Vilchinskaya, N. , Krivoi, I. , & Shenkman, B. (2018). AMP‐activated protein kinase as a key trigger for the disuse‐induced skeletal muscle remodeling. International Journal of Molecular Sciences, 19, 3558. 10.3390/ijms19113558 30424476 PMC6274864

[phy270602-bib-0082] Waisman, A. , Norris, A. M. , Elías Costa, M. , & Kopinke, D. (2021). Automatic and unbiased segmentation and quantification of myofibers in skeletal muscle. Scientific Reports, 11, 11793. 10.1038/s41598-021-91191-6 34083673 PMC8175575

[phy270602-bib-0083] Waki, H. , Yamauchi, T. , Kamon, J. , Kita, S. , Ito, Y. , Hada, Y. , Uchida, S. , Tsuchida, A. , Takekawa, S. , & Kadowaki, T. (2005). Generation of globular fragment of adiponectin by leukocyte elastase secreted by monocytic cell line THP‐1. Endocrinology, 146, 790–796. 10.1210/en.2004-1096 15528304

[phy270602-bib-0084] Walowski, C. O. , Herpich, C. , Enderle, J. , Braun, W. , Both, M. , Hasler, M. , Müller, M. J. , Norman, K. , & Bosy‐Westphal, A. (2023). Analysis of the adiponectin paradox in healthy older people. Journal of Cachexia, Sarcopenia and Muscle, 14, 270–278. 10.1002/jcsm.13127 36401062 PMC9891976

[phy270602-bib-0085] Wang, J. , Wang, F. , Zhang, P. , Liu, H. , He, J. , Zhang, C. , Fan, M. , & Chen, X. (2017). PGC‐1α over‐expression suppresses the skeletal muscle atrophy and myofiber‐type composition during hindlimb unloading. Bioscience, Biotechnology, and Biochemistry, 81, 500–513. 10.1080/09168451.2016.1254531 27869526

[phy270602-bib-0086] Wang, Y. , Lam, K. S. L. , Yau, M. , & Xu, A. (2008). Post‐translational modifications of adiponectin: Mechanisms and functional implications. The Biochemical Journal, 409, 623–633. 10.1042/BJ20071492 18177270

[phy270602-bib-0087] Wang, Y. , & Pessin, J. E. (2013). Mechanisms for fiber‐type specificity of skeletal muscle atrophy. Current Opinion in Clinical Nutrition and Metabolic Care, 16, 243–250. 10.1097/MCO.0b013e328360272d 23493017 PMC4327989

[phy270602-bib-0088] Warneke, K. , Lohmann, L. H. , Lima, C. D. , Hollander, K. , Konrad, A. , Zech, A. , Nakamura, M. , Wirth, K. , Keiner, M. , & Behm, D. G. (2023). Physiology of stretch‐mediated hypertrophy and strength increases: A narrative review. Sports Medicine, 53, 2055–2075. 10.1007/s40279-023-01898-x 37556026 PMC10587333

[phy270602-bib-0089] Yamauchi, T. , Iwabu, M. , Okada‐Iwabu, M. , & Kadowaki, T. (2014). Adiponectin receptors: A review of their structure, function and how they work. Best Practice & Research Clinical Endocrinology & Metabolism, 28, 15–23. 10.1016/j.beem.2013.09.003 24417942

[phy270602-bib-0090] Yamauchi, T. , & Kadowaki, T. (2013). Adiponectin receptor as a key player in healthy longevity and obesity‐related diseases. Cell Metabolism, 17, 185–196. 10.1016/j.cmet.2013.01.001 23352188

[phy270602-bib-0091] Yamauchi, T. , Kamon, J. , Ito, Y. , Tsuchida, A. , Yokomizo, T. , Kita, S. , Sugiyama, T. , Miyagishi, M. , Hara, K. , Tsunoda, M. , Murakami, K. , Ohteki, T. , Uchida, S. , Takekawa, S. , Waki, H. , Tsuno, N. H. , Shibata, Y. , Terauchi, Y. , Froguel, P. , … Kadowaki, T. (2003). Cloning of adiponectin receptors that mediate antidiabetic metabolic effects. Nature, 423, 762–769. 10.1038/nature01705 12802337

[phy270602-bib-0092] Yamauchi, T. , Nio, Y. , Maki, T. , Kobayashi, M. , Takazawa, T. , Iwabu, M. , Okada‐Iwabu, M. , Kawamoto, S. , Kubota, N. , Kubota, T. , Ito, Y. , Kamon, J. , Tsuchida, A. , Kumagai, K. , Kozono, H. , Hada, Y. , Ogata, H. , Tokuyama, K. , Tsunoda, M. , … Kadowaki, T. (2007). Targeted disruption of AdipoR1 and AdipoR2 causes abrogation of adiponectin binding and metabolic actions. Nature Medicine, 13, 332–339. 10.1038/nm1557 17268472

[phy270602-bib-0093] Zhang, M. Y. , Zhang, W. J. , & Medler, S. (2010). The continuum of hybrid IIX/IIB fibers in normal mouse muscles: MHC isoform proportions and spatial distribution within single fibers. American Journal of Physiology. Regulatory, Integrative and Comparative Physiology, 299, R1582–R1591. 10.1152/ajpregu.00402.2010 20861278 PMC3007186

