# Supplementary material for: Disuse‐induced muscle‐type specific alterations and adiponectin pathway response in male mice
Source: Physiol Rep. 2025 Oct 20;13(20):e70602. doi: 10.14814/phy2.70602 (PMC12538006; doi:10.14814/phy2.70602)
Supplement: Supplementary file 1 — Table S1. Primers used in RTqPCR analyses. [file PHY2-13-e70602-s007.docx]

**Suppl. Table S1.** Mouse primers used in RTqPCR analysis.

| **Gene** | **Sequence** | **Tm** |
| --- | --- | --- |
| ***Rplp0*** | Fw: GGA-CCC-GAG-AAG-ACC-TCC-TT  Rv: GCA-CAT-CAC-TCA-GAA-TTT-CAA-TGG | 60°C |
| ***Adipoq*** | Fw: GTT-GCA-AGC-TCT-CCT-GTT-CC  Rv: TCT-CCA-GGA-GTG-CCA-TCT-CT | 58°C |
| ***Adipor1*** | Fw: TCT-TCG-GGA-TGT-TCT-TCC-TGG  Rv: TTT-GGA-AAA-AGT-CCG-AGA-GAC-C | 62°C |
| ***Adipor2*** | Fw: CCT-TTC-GGG-CCT-GTT-TTA-AGA  Rv: GAG-TGG-CAG-TAC-ACC-GTG-TG | 62°C |
| ***Cdh13*** | Fw: GCC-CTC-GTG-AGC-CTT-CTT-C  Rv: CAC-CCT-GAG-GTC-CGT-GAT-GT | 58°C |
| ***Fbxo32*** | Fw:GCA-AAC-ACT-GCC-ACA-TTC-TCT-C  Rv: CTT-GAG-GGG-AAA-GTG-AGA-CG | 60°C |
