# Supplementary figures and images for: Disuse‐induced muscle‐type specific alterations and adiponectin pathway response in male mice
Source: Physiol Rep. 2025 Oct 20;13(20):e70602. doi: 10.14814/phy2.70602 (PMC12538006; doi:10.14814/phy2.70602)

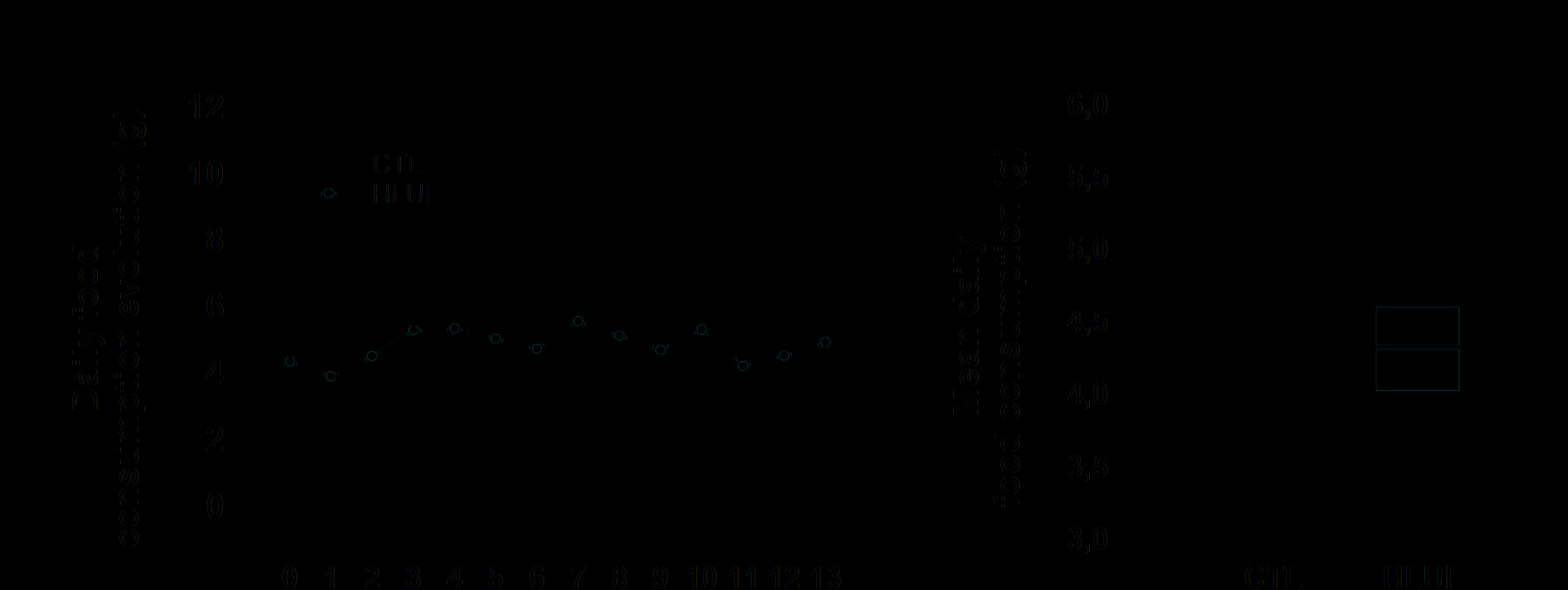

Supplement: Supplementary file 2 — Figure S1. Food consumption in CTL and HLUI mice. (A) Food consumption was measured daily. Data plotted as mean ± SD and compared using a Two‐way ANOVA repeated measures (p < 0.001, HLUI vs. CTL). (B) Mean daily food consumption. Data presented as boxplots; groups compared using a Student’s t‐test (NS). CTL group: N = 10, HLUI group: N = 11. [file PHY2-13-e70602-s009.tif]

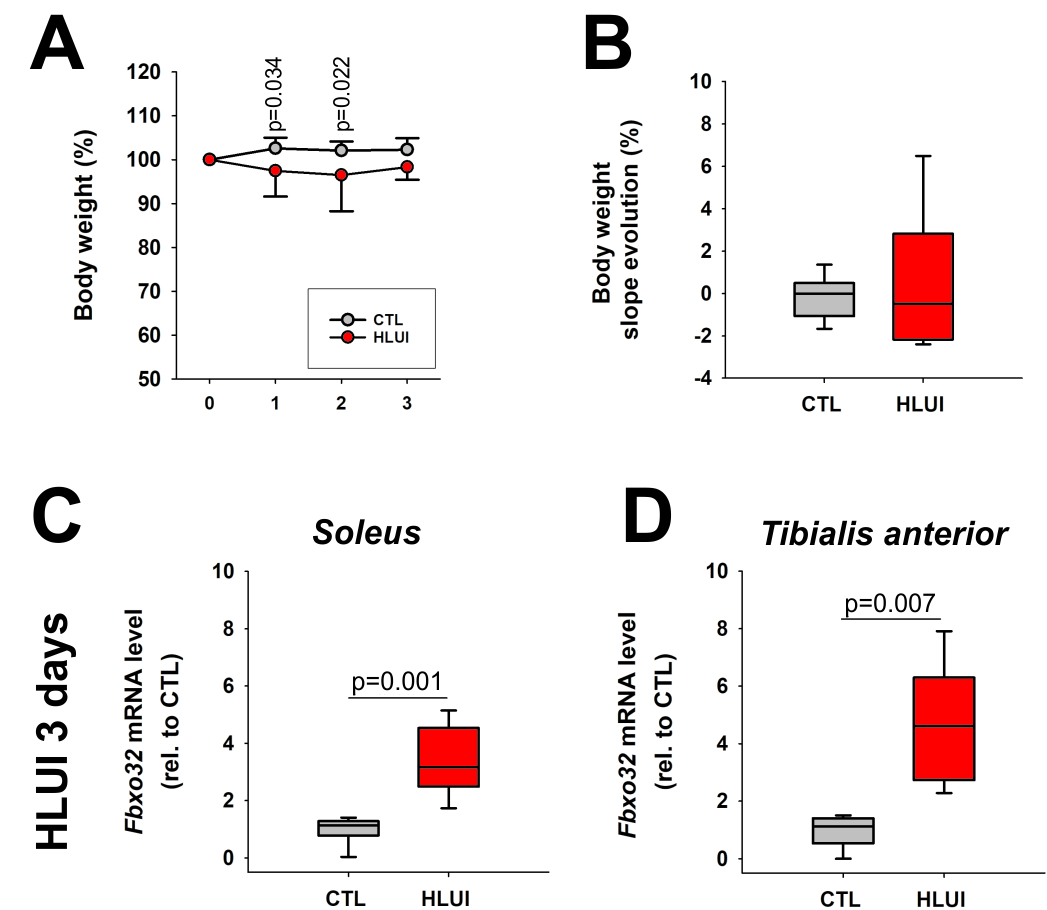

Supplement: Supplementary file 3 — Figure S2. Early effects of HLUI (day 3) on mouse bodyweight and Fbxo 32 expression in Soleus and Tibialis anterior muscles. (A) Daily measurements normalized to b.w. at D0 (defined as the 100% baseline). Data presented as mean ± SD and groups compared using a Two‐way ANOVA repeated measures (p = 0.034, p = 0.022, as indicated). (B) Fbxo32 mRNA level was assessed in (C) the Soleus and (D) the Tibialis anterior muscles by RTqPCR. Data presented as boxplot; groups compared using a Student’s t‐test (p = 0.001, p = 0.007, as indicated). CTL group: N = 6, HLUI group: N = 6. [file PHY2-13-e70602-s008.jpg]

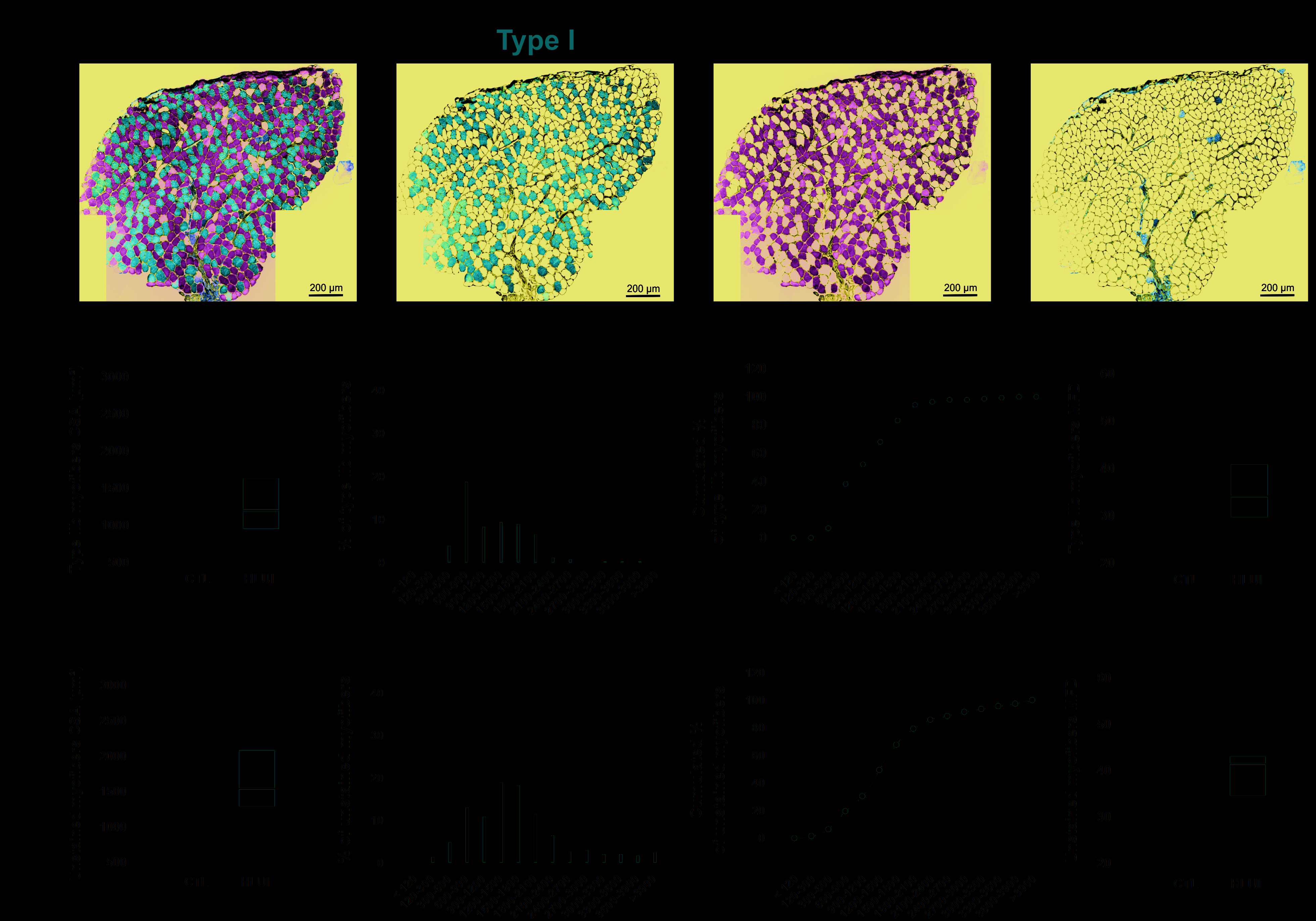

Supplement: Supplementary file 4 — Figure S3. Effects of HLUI on type IIb and unstained (IIx) myofibers in the Soleus muscle. (A) Representative fields. (B–C) Cross‐sectional Area (CSA), myofiber CSA distribution, and Minimum Feret’s Diameter (MFD) in type IIb (B) and unstained (C) myofibers. Data presented as in Fig.2. CSA: Student’s t‐test (NS). Myofiber CSA distribution: Statistical tests were not performed because of the scarcity of type IIb fibers in the Soleus muscle. For unstained myofibers, groups were compared using a Chi‐square (Chi2) test (NS). MFD: Student’s t‐test (p = 0.026, as indicated). CTL group: N = 5, HLUI group: N = 5. [file PHY2-13-e70602-s005.tif]

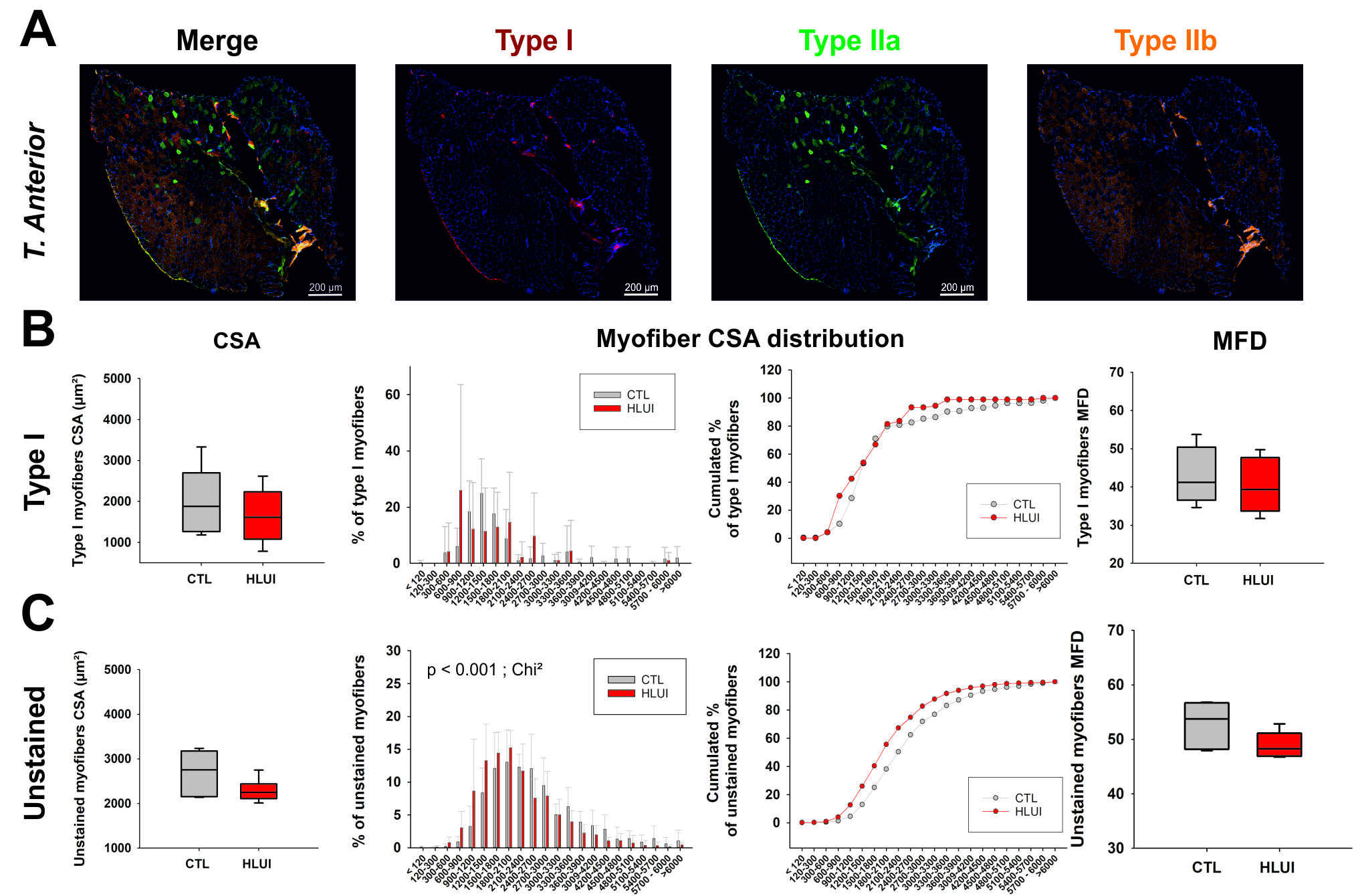

Supplement: Supplementary file 5 — Figure S4. Effects of HLUI on type I and unstained (IIx) myofibers in the Tibialis anterior muscle. (A) Representative fields. (B‐C) Cross‐sectional Area (CSA), myofiber CSA distribution, and Minimum Feret’s Diameter (MFD) in type I (B) and unstained myofibers (C). Data presented as in Fig.2. CSA: Student’s t‐test in type I myofibers; Welch’s t‐test in unstained myofibers (NS). Myofiber CSA distribution: Chi‐square (Chi2) tests (p<0.001, as indicated). MFD: Student’s t‐test in type I myofibers, Welch’s t‐test in unstained myofibers (NS). CTL group: N = 5, HLUI group: N = 5. [file PHY2-13-e70602-s006.jpg]

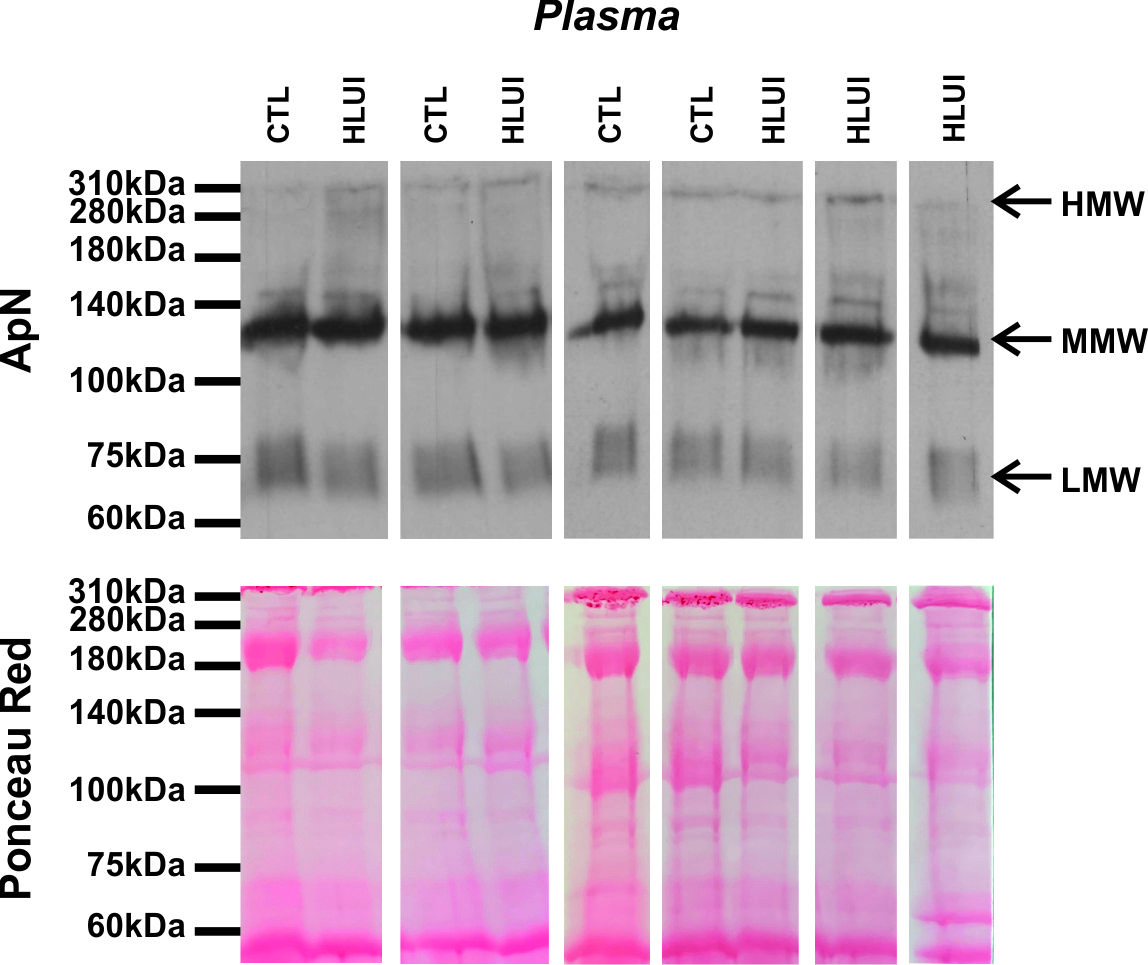

Supplement: Supplementary file 6 — Figure S5. Adiponectin circulating forms: western blot immunodetection and corresponding Ponceau Red. [file PHY2-13-e70602-s010.jpg]

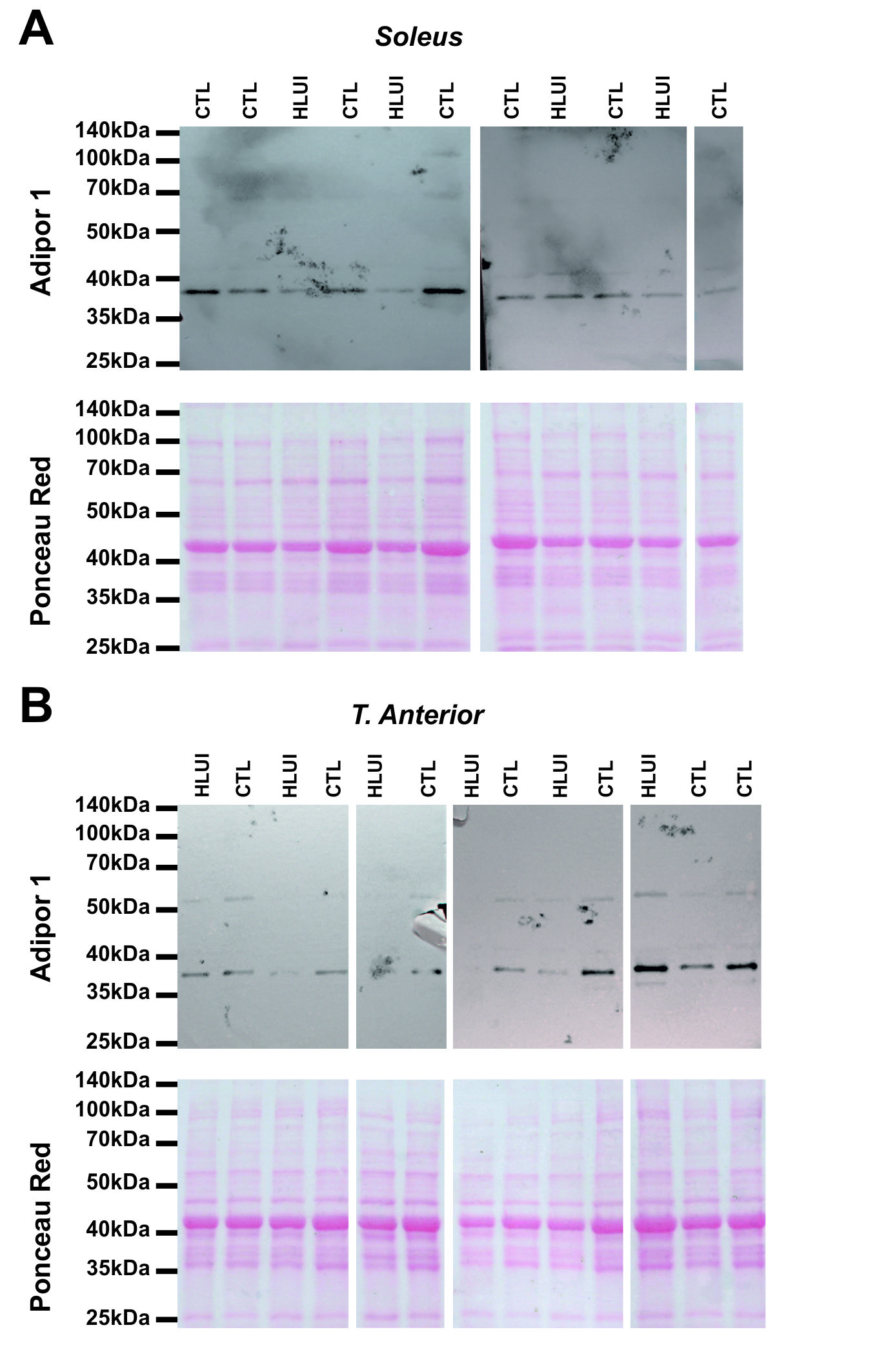

Supplement: Supplementary file 7 — Figure S6. Adipor1: western blot immunodetection and corresponding Ponceau Red in (A) Soleus and (B) Tibialis anterior muscles. [file PHY2-13-e70602-s001.jpg]

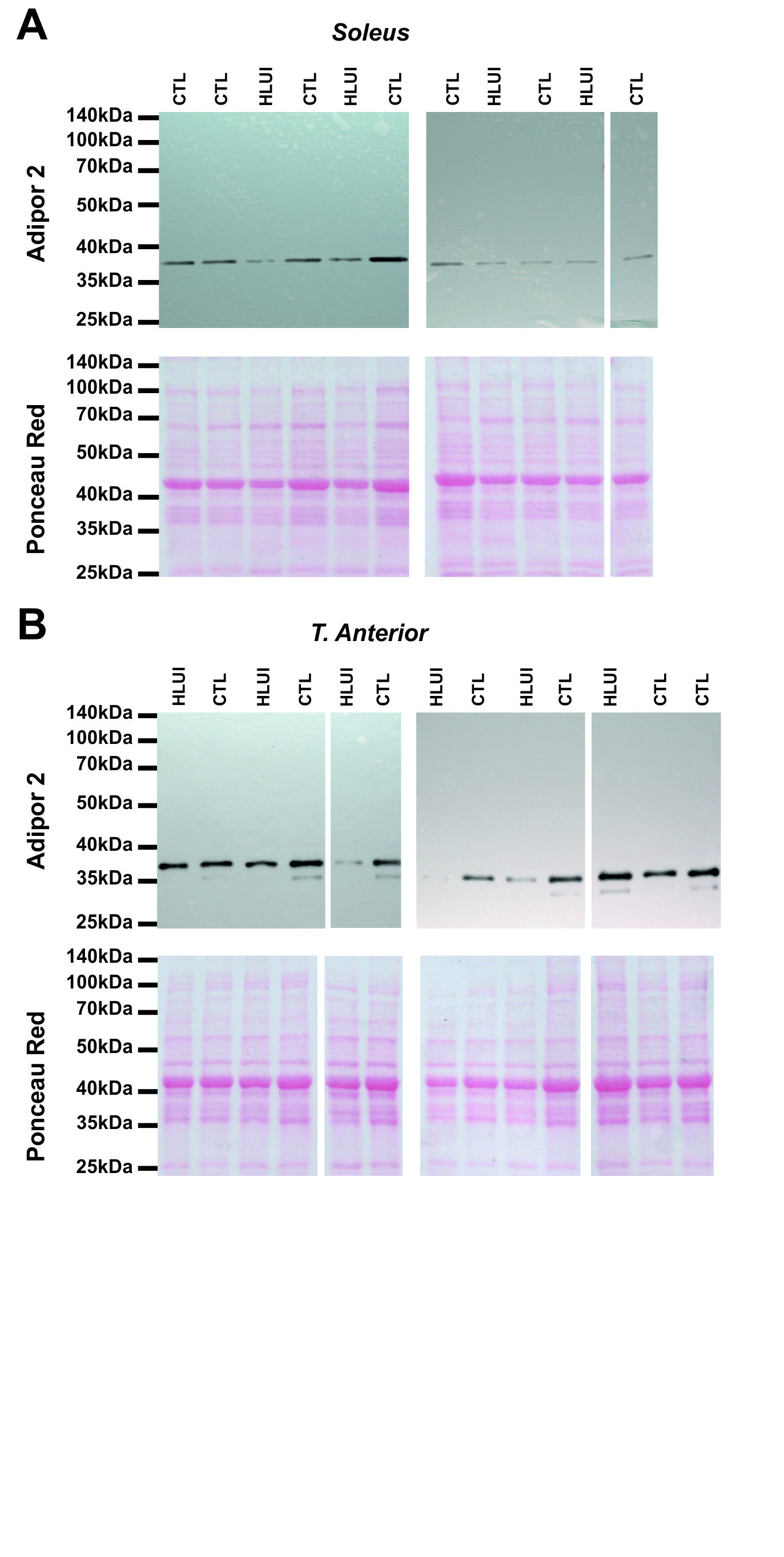

Supplement: Supplementary file 8 — Figure S7. Adipor2: western blot immunodetection and corresponding Ponceau Red in (A) Soleus and (B) Tibialis anterior muscles. [file PHY2-13-e70602-s003.jpg]

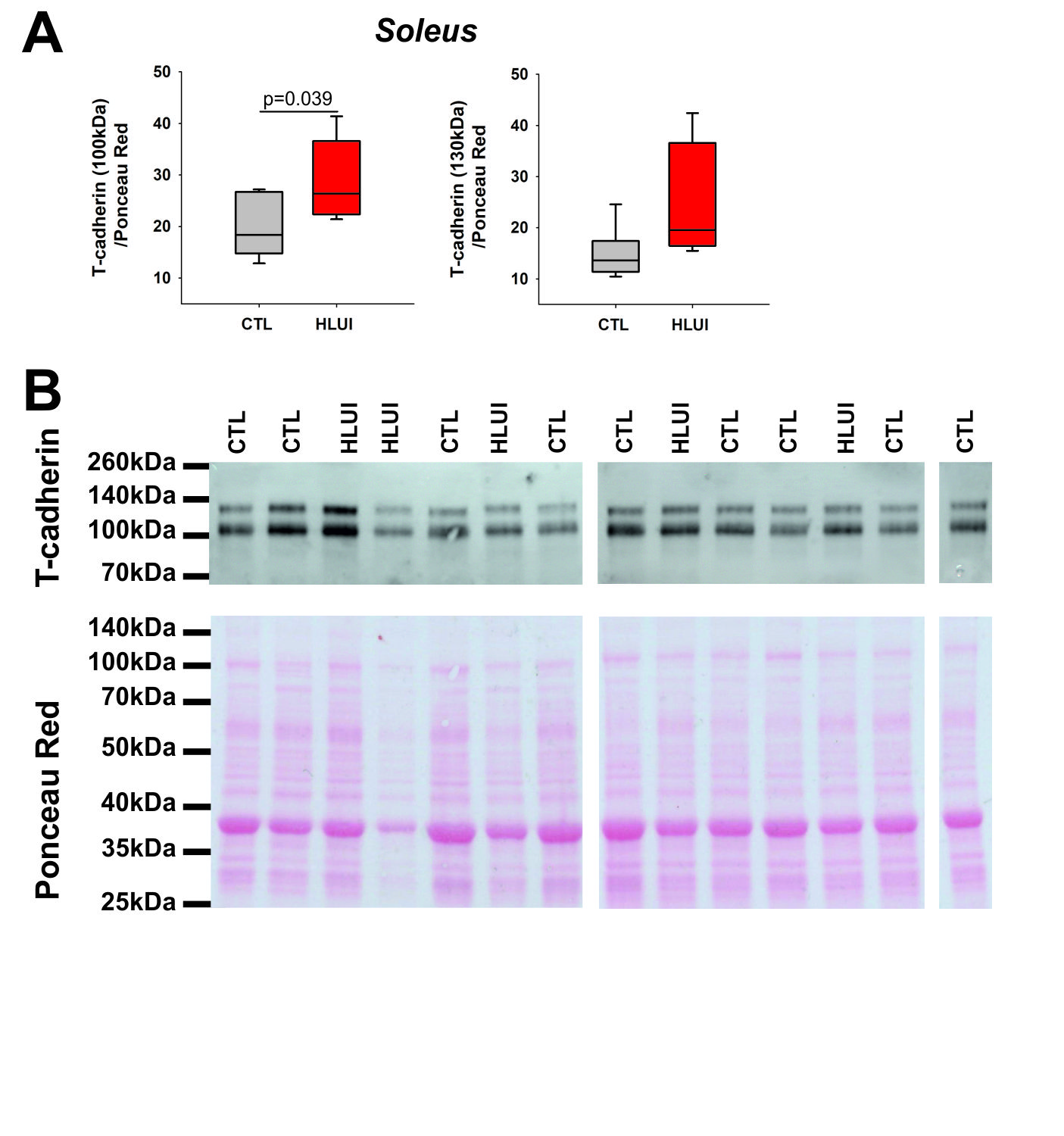

Supplement: Supplementary file 9 — Figure S8. Effect of HLUI on mature (100kDa) and pro‐domain bearing (130kDa) T‐cadherin protein levels in the Soleus muscle. (A) T‐cadherin protein levels were determined using PAGE‐SDS and western blot. Densitometric signal normalized to Ponceau Red. Data presented as boxplots; groups compared using Student’s t‐tests (p = 0,039, as indicated). CTL group: N = 7, HLUI group: N = 5. (B) Representative blots. [file PHY2-13-e70602-s002.jpg]

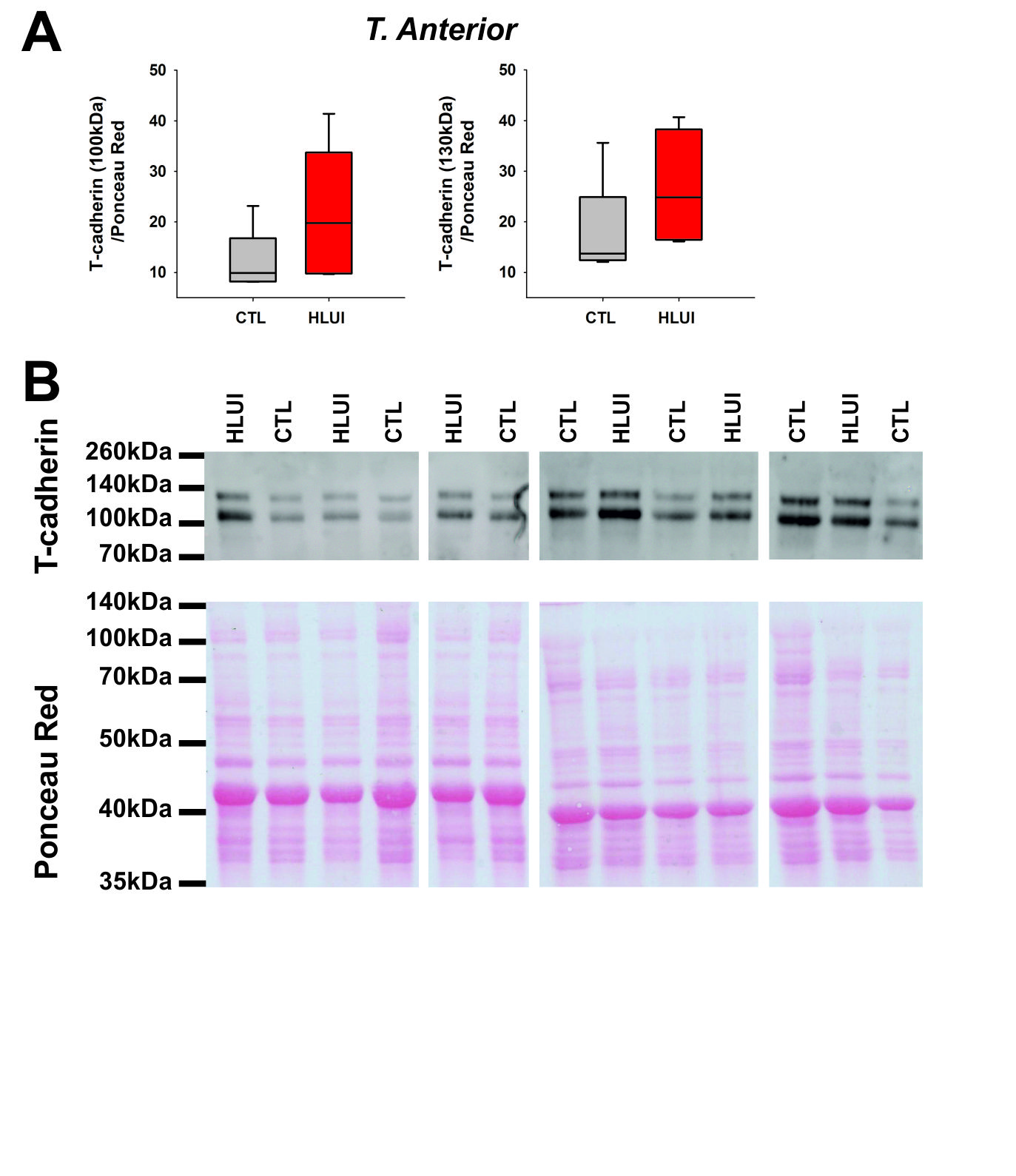

Supplement: Supplementary file 10 — Figure S9. Effect of HLUI on mature (100kDa) and pro‐domain bearing (130kDa) T‐cadherin protein level in the Tibialis anterior muscle. (A) T‐cadherin protein levels were determined using PAGE‐SDS and western blot. Densitometric signal normalized to Ponceau Red. Data presented as boxplot; groups compared using Student’s t‐tests (NS). CTL group: N = 7, HLUI group: N = 5. (B) Representative blots. [file PHY2-13-e70602-s004.jpg]
